# Novel Arf1 Inhibitors Drive Cancer Stem Cell Aging and Potentiate Anti‐Tumor Immunity

**DOI:** 10.1002/advs.202404442

**Published:** 2024-09-03

**Authors:** Yuetong Wang, Qiaoming Li, Yahui Ding, Chenfei Luo, Jun Yang, Na Wang, Ning Jiang, Tiange Yao, Guohao Wang, Guoming Shi, Steven X. Hou

**Affiliations:** ^1^ Department of Cell and Developmental Biology at School of Life Sciences State Key Laboratory of Genetic Engineering Institute of Metabolism and Integrative Biology Human Phenome Institute Department of Liver Surgery and Transplantation of Liver Cancer Institute at Zhongshan Hospital Fudan University Shanghai 200438 China; ^2^ The Basic Research Laboratory Center for Cancer Research National Cancer Institute at Frederick National Institutes of Health Frederick MD 21702 USA; ^3^ Leading Contact

**Keywords:** anti‐tumor immunity, Arf1 inhibitor, cancer stem cell, superior T cells, trans‐cellular signaling

## Abstract

The small G protein Arf1 has been identified as playing a selective role in supporting cancer stem cells (CSCs), making it an attractive target for cancer therapy. However, the current Arf1 inhibitors have limited translational potential due to their high toxicity and low specificity. In this study, two new potent small‐molecule inhibitors of Arf1, identified as DU101 and DU102, for cancer therapy are introduced. Preclinical tumor models demonstrate that these inhibitors triggered a cascade of aging in CSCs and enhance anti‐tumor immunity in mouse cancer and PDX models. Through single‐cell sequencing, the remodeling of the tumor immune microenvironment induced by these new Arf1 inhibitors is analyzed and an increase in tumor‐associated CD8+ CD4+ double‐positive T (DPT) cells is identified. These DPT cells exhibit superior features of active CD8 single‐positive T cells and a higher percentage of TCF1+PD‐1+, characteristic of stem‐like T cells. The frequency of tumor‐infiltrating stem‐like DPT cells correlates with better disease‐free survival (DFS) in cancer patients, indicating that these inhibitors may offer a novel cancer immunotherapy strategy by converting the cold tumor immune microenvironment into a hot one, thus expanding the potential for immunotherapy in cancer patients.

## Graphical abstract

Potent small‐molecule inhibitors of Arf1, designated as DU101 and DU102, have been discovered to initiate a series of aging processes in CSCs and systemically remodeled immune microenvironment including increasing the population of tumor‐associated CD8+ CD4+ double‐positive T (DPT) cells in preclinical mouse cancer and PDX models.

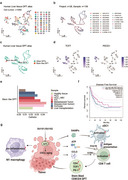

## 1 Introduction

The small G protein Arf1, a member of the ADP‐ribosylation factor (Arf) small G protein family, is localized at the interface of multiple organelles. Its role involves coordinating organelle communication, fatty acid metabolism, and maintaining mitochondrial homeostasis.^[^ ^1^ ^]^ Research has revealed high expression of Arf1 in various types of human cancers, including but not limited to breast, hepatocellular, colon, and prostate cancer.^[^ ^2^, ^3^ ^]^ And, elevated Arf1 expression is inversely linked to the prognosis of individuals diagnosed with cancer.^[^ ^2^, ^4^, ^5^ ^]^

Our recent study has revealed that the Arf1‐regulated lipid metabolism plays a crucial role in sustaining the homeostasis of cancer stem cells and neurons selectively.^[^ ^2^, ^6^, ^7^, ^8^, ^9^, ^10^ ^]^ In Drosophila, the knockout of Arf1 in differentiated cells of the intestine and Malpighian Tubules (kidney) yields no discernible phenotype. However, the knockout of Arf1 in stem cells triggers a cascade of cellular organelle aging (exhibiting hallmarks of aging),^[^ ^11^, ^12^, ^13^ ^]^ including lipid droplet (LD) accumulation, mitochondrial damage, reactive oxygen species (ROS) production, endoplasmic reticulum (ER) stress, and lysosomal protein aggregation. Abnormal stem cells in Drosophila melanogaster activate neighboring enterocyte (EC) cells by transmitting danger signals, resulting in the eradication of damaged stem cells through a process of killing, engulfing, and removal.^[^ ^6^, ^7^ ^]^ In mice, knockout of Arf1 in hepatocytes with Albumin‐Cre (Alb‐Cre) or normal adult intestinal stem cells with Lgr5‐CreER does not have any phenotype, while knocking down Arf1 in cancer stem cells (CSCs) of the intestine with Lgr5‐CreER or of the liver with Axin2‐CreER elicits a similar organelle aging cascade.^[^ ^2^ ^]^ The abnormal CSCs then send danger signals to activate antigen‐presenting dendritic cells (DCs) in the tumor immune microenvironment.^[^ ^2^, ^14^ ^]^ In contrast to current therapies, Arf1‐targeted therapy offers a dual anticancer mechanism by promoting CSC aging and enhancing the anti‐tumor activity of immune cells. However, further investigation is required to understand the anti‐tumor immunity response regulated by Arf1‐targeted therapy.

The high toxicity and low specificity of the existing Arf1 inhibitors, such as Golgicide A (GCA), Brefeldin A (BFA), and Exo2 have greatly limited their translational potential and related research.^[^ ^15^, ^16^, ^17^, ^18^ ^]^ Genetic screens in model organisms have been extensively used to study gene's biological functions.^[^ ^19^ ^]^ To date, the utilization of such screens for the identification of target genes associated with tumor immunity has been limited. Our prior investigations demonstrated the selective eradication of RasV12‐transformed stem cell tumors in *Drosophila* Malpighian tubules (MT, fly kidney) through necrosis upon the knockdown of the COPI/Arf1‐lipolysis pathway. This process preserved differentiated cells. Notably, administering existing Arf1 inhibitors to flies resulted in the targeted elimination of tumorigenic stem cells while leaving normal stem cells unharmed.^[^ ^6^, ^7^ ^]^

Here, in the Drosophila stem cell tumor system, a screening of over 100 chemicals, modified based on Exo2, led to the discovery of two new potent small‐molecule inhibitors of Arf1 for cancer therapy, denoted as DU101 and DU102. Demonstrations indicated that these newly designed Arf1 inhibitors exhibit low toxicity and robust anti‐tumor activities in various mouse tumor models, including liver, colon, breast, and melanoma, as well as PDX tumor models. Treatment with the inhibitors notably augmented T cell tumor infiltration, augmented the activation of CD8+ T cells via ATP, and induced the formation of tumor‐associated PD‐1+ TCF1+ CD8+ CD4+ stem‐like DPT cells. Consequently, the new Arf1 inhibitors present an auspicious strategy for cancer immunotherapy and are presently under evaluation for clinical trials in patients with advanced solid tumors.

## 2 Results

### 2.1 Discovery of the Novel Arf1 Inhibitors

**Figure 1:**
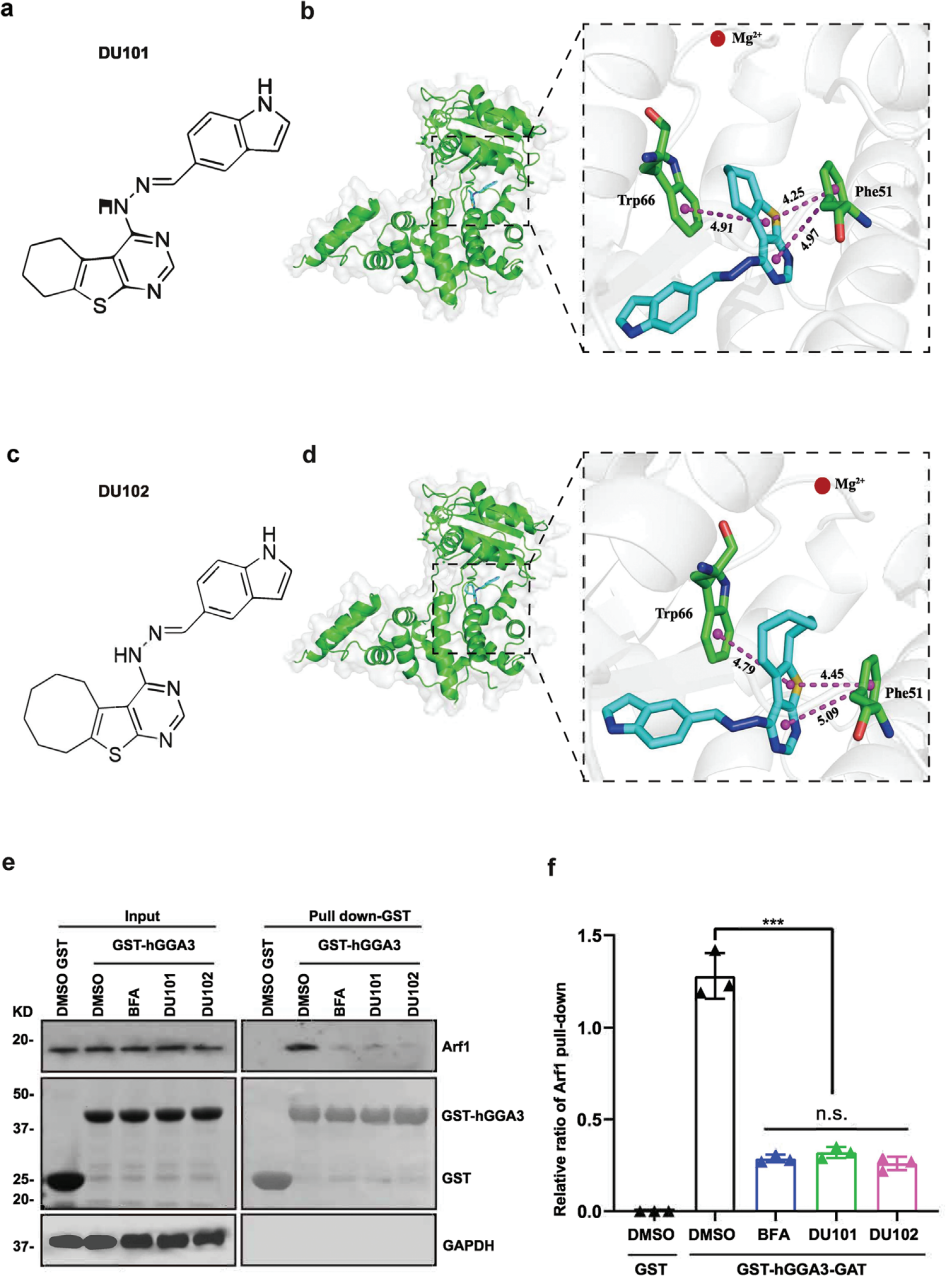
DU101 and DU102 are new potent inhibitors of Arf1. Chemical structure of the compound DU101. Representation of DU101 docking onto a published Sec7 domain of the Arf1‐GEF cocrystal structure (PDB ID 1RE0, 2.40 Å). (c) Chemical structure of the compound DU102. (d) Representation of DU102 docking onto a published Sec7 domain of the Arf1‐GEF cocrystal structure. (e) DU101 and DU102 caused a decrease in GBF1‐dependent Arf1 activation. Huh7 cells were treated with DMSO, BFA, DU101 or DU102. Arf1 activities were assessed by GGA3 pull‐down and western blotting assays. (f) Quantification of the data in (e). Data are shown as the mean ± SEM. Student's *t*‐test. ***p < 0.001; n.s., not significant. BFA, brefeldin A.

Using a *Drosophila* stem cell tumor system, we screened and discovered two new potent small‐molecule inhibitors of Arf1, DU101, and DU102 (**Figure** **1**a–d; Figure S1a–c, Supporting Information details are described in STAR methods), which specifically and effectively killed the cancer stem cells. We first validated the biochemical and biophysical effects of DU101 and DU102 on Arf1. It was reported that the Sec7 domain of Golgi‐associated Arf‐GEF (GBF1) interacts with GDP‐bound Arf1 to regulate GDP‐GTP exchange for Arf1 activation.^[^ ^20^ ^]^ We performed high‐precision molecular docking analysis and found that both DU101 and DU102 bound to the catalytic pocket of the Arf1‐GDP‐Sec7 (PDB code: 1RE0) complex at 2.40 Å (Figure 1b,d). We then directly examined the binding efficiency of DU101 or DU102 to the Sec7 domain of GBF1 by isothermal titration calorimetry and found that both DU101 and DU102 effectively bound the purified Arf1‐GDP‐Sec7 domain (Figure S2a,b, Supporting Information). Drug affinity responsive target stability (DARTS) is applied to track and identify target proteins based on the stability changes when the target protein binds to small molecules.^[^ ^21^ ^]^ By DARTS, we showed that DU101 and DU102 could significantly enhance the stability of Arf1 in a dosage‐dependent way. As shown in Figure S2c,d (Supporting Information), only the protein level of Arf1 increased in cells treated with proteinase combined with DU101 or DU102 indicating a specific protecting effect of Arf1 stability of these inhibitors. While the other ARF isoforms we tested, including Arf3, Arf5, Arf6, and pan‐Ras, the protein levels showed no obvious difference compared control group with DU101 or DU102 treatment group in cells with proteinase treatment (Figure S2c,d, Supporting Information). For the Arf1 activity assay, the GST‐GGA3‐PBD fusion protein selectively binds the activated form of GTP‐bound Arf1 and Arf1 activity in cells can be examined by pulling down GST‐hGGA3‐Arf1.^[^ ^22^ ^]^ We found that DU101 or DU102 treatment significantly inhibited Arf1 activity (Figure 1e,f). These results implied that our novel Arf1 inhibitor could inhibit Arf1 activity by binding to Arf1 and Arf‐GEF.

To evaluate the cell toxicity of DU101 and DU102, we examined the cell viability of the normal stem cells in *Drosophila*, as well as CT26, 4T1, B16‐F10, and LLC cells after treatments with DMSO and DU101 or DU102, and found that DU101 and DU102 exhibited low cell toxicity (Figures S1b and S2e–h, Supporting Information). To evaluate the toxicity in vivo, we compared different organs from mice with or without DU102 treatment. By hematoxylin‐eosin (HE) staining, no significant histomorphology alterations were discovered (Figure S2i, Supporting Information). Together, these results demonstrated that the two new Arf1 inhibitors, DU101 and DU102, have low toxicity, and are potentially effective Arf1 inhibitors.

### 2.2 The New Arf1 Inhibitors Induced Tumor Regression and Prolonged Lifespan in Multiple Mouse and PDX Tumor Models

**Figure 2:**
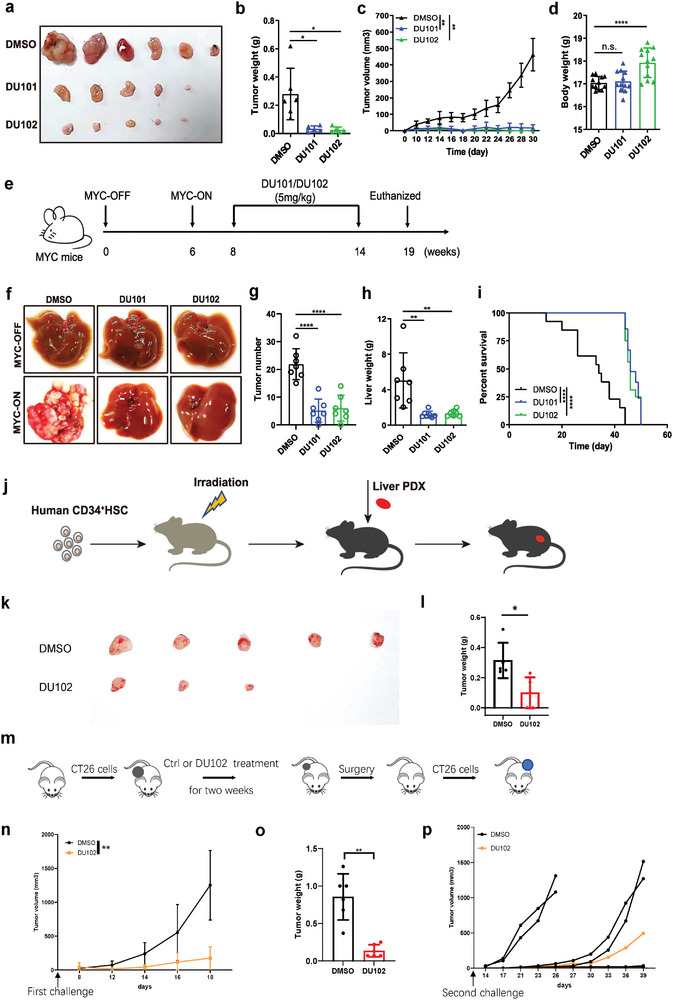
DU101 and DU102 induced tumor regression and prolonged lifespan in mouse and PDX liver tumor models. a) Images of tumor sizes from Hepa1‐6 cells transplanted into C57BL/6J mice and treated with the indicated reagents. b–d) Bar graphs of tumor weights (b), curves of tumor volumes (c), and bar graphs of the body weight of mice described in (a). (n = 6 mice in each group). e) Experimental setup for the MYC‐ON mice. f–i) Representative liver images of MYC‐OFF or MYC‐ON mice treated with DMSO, DU101, or DU102 (f), liver surface tumor counts (g), liver weights (h), and survival curves (i) of MYC‐ON mice with the indicated treatments. (n = 10 mice in each group). j) Experimental setup for generating humanized immune and liver tumor PDX mice. k–l) Representative tumor images (k) and tumor weights (l) of humanized immune and liver tumor PDX mice treated with the indicated reagents. (n = 5 mice in each group). m) Experimental setup for the second tumor challenge experiment. n‐o) Representative tumor growth curve (n) and tumor weights (o) of mice with the first CT26 tumor cell challenge treated with the indicated reagents. (n = 5 mice in each group). p) Tumor growth curve for each mouse with second CT26 tumor cell challenge. (n = 5 mice in each group). Data are shown as the mean ± SEM. Student's *t*‐test, D‐Kaplan‒Meier, and log‐rank test (i). *p < 0.05, **p < 0.01, ****p < 0.0001; n.s., not significant.

To investigate the therapeutic effect of DU101 and DU102 on tumors, we generated allografts of mouse liver cancer Hepa1‐6 cells in C57 mice and then treated the mice with DMSO, DU101 or DU102, respectively. We observed dramatically reduced tumor size and volume (**Figure** **2**a–c) but no decrease in body weight (Figure 2d) in mice treated with DU101 and DU102 at a dose of 5 mg kg^−1^ for 2 weeks by gavage in comparison with mice treated with DMSO.

We further examined the anti‐tumor effect of DU101 and DU102 in our previously used liver CSC transgenic mouse model,^[^ ^2^ ^]^ Axin2‐CreER driven MYC‐ON liver tumor mouse model. MYC‐ON mice were switched to a normal diet to induce tumor formation at the age of 6 weeks and then treated with DMSO, DU101, or DU102 at the dose of 5 mg kg^−1^ by gavage for 6 weeks (Figure 2e). We found that DU101 and DU102 treatment significantly reduced liver tumors (Figure 2f–h) and extended the lifespan of the MYC‐ON mice (Figure 2i) in comparison with DMSO treatment.

To exam the general application of these inhibitors, we also treated allografts of mouse colon cancer CT‐26 cells, mouse breast cancer 4T1 cells, and mouse melanoma B16‐F10 cells with DMSO, DU101 or DU102, which contain 98.3%, 40.1% and 76.6% of the CD133^+^CD44^hi^ cells (potential CSC markers) respectively.^[^ ^2^ ^]^ We found that DU101 or DU102 treatment also dramatically regressed tumors but had no effects on mouse body weights in comparison with mice treated with DMSO in the three mouse allograft tumor models (Figure S3a–i, Supporting Information).

To investigate the anti‐tumor effect of the new Arf1 inhibitors on human tumors, we seeded mouse livers with fresh human liver tumor samples and generated liver cancer patient‐derived xenografts (PDXs) in humanized HSC‐NCG mice (Figure 2j), which retained the major characteristics of the original human tumors and tumor immune microenvironment. We then treated humanized mice with DMSO or DU102 at 5 mg kg^−1^ by oral gavage for one week. In comparison with DMSO treatment, we found that DU102 treatment significantly reduced the tumor sizes of liver PDX (Figure 2k–l; Figure S4a, Supporting Information) but had no impact on the mouse body weights (Figure S4b, Supporting Information) in the humanized HSC‐NCG mice.

To test whether DU102 treatment generated immune memory, we performed a second tumor challenge in DU102‐treated mice as the scheme showed (Figure 2m). Our results showed that DU102 treatment could effectively inhibit tumor growth after the first tumor challenge (Figure 2n–o). After the excision of the tumor, a second tumor challenge was performed. Mice treated with DU102 have a low tumorigenesis rate (1 in 5 compared with 4 in 5 Figure 2p). Thus, the DU102 treatment supports immune memory.

To demonstrate the specificity of DU101 and DU102, we treated BALB/c mice with allograft tumors from the Arf1‐deficient CT26 cells (Figure S4c–e, Supporting Information), control CT26 cells (Figure S4f,j, Supporting Information), Arf4‐deficient CT26 cells (Figure S4g,h,l, Supporting Information), Arf5‐deficient CT26 cells (Figure S4g,i,m, Supporting Information) and Arf6‐deficient CT26 cells (Figure S4g,j,n, Supporting Information). DU101 or DU102 treatment did not further affect tumor and body weights in the Arf1‐deficient CT26 cell tumor‐bearing mice, indicating that DU101 and DU102 were specific Arf1 inhibitors. While, in other groups, DU102 treatment could still effectively inhibit tumor growth. Collectively, the above results demonstrated that the new Arf1‐specific inhibitors DU101 and DU102 could significantly regress tumors with minimal toxic effects in multiple mouse and liver PDX models. Thus, the inhibitors might have great translational potential.

### 2.3 The New Arf1 Inhibitors Induced Tumor Cell Aging Cascade

We previously reported that genetic ablation of *Arf1* in *Drosophila* stem cells and mouse CSCs resulted in cell aging cascade,^[^ ^2^, ^6^, ^7^, ^14^ ^]^ including LD accumulation, ROS production, mitochondrial damage, ER stress, and lysosomal protein aggregation. We then compared the effect of Arf1 inhibition to genetic deletion and found that treatment of CT26 cells with DU101 and DU102 dramatically reduced the mitochondrial membrane potential (Figure S5a,b, Supporting Information), increased ROS production as assayed by elevated Mitosox level (Figure S5c,d, Supporting Information), induced expression of the lysosome protein LAMP1 (Figure S5c,d, Supporting Information), and increased protein aggregation as illustrated by increased staining of Proteostat dye (Figure S5e,f, Supporting Information), in comparison with those in CT26 cells treated with DMSO control. The cellular organelle aging cascade triggered by Arf1 knockout has some similarities with commonly known cellular senescence.^[^ ^23^ ^]^ Senescence‐associated β‐galactosidase (SA‐β‐gal) is a molecular marker of senescent cells.^[^ ^24^ ^]^ We stained SA‐β‐gal and did not find a change in cells treated with the Arf1 inhibitors in comparison with cells treated with DMSO (Figure S5g,h, Supporting Information). These data suggest that, consistent with previously reported effects of genetic ablation of *Arf1*, the new Arf1 inhibitors DU101 and DU102 also promote cell aging cascade, which might be different from the commonly known cellular senescence.

### 2.4 The New Arf1 Inhibitors Promoted T Cell Infiltration, Activation, and Memory T Cell Population

In the previous study, we found that knocking down the Arf1 pathway in mouse CSCs with Lgr5‐CreER and Axin2‐CreER activated a systemic anti‐tumor immune response to destroy tumors.^[^ ^2^ ^]^ To demonstrate that the antitumor activity of DU101 and DU102 specifically relies on immune response, we first treated nude mice with allograft tumors from CT26 cells and found that DU101 or DU102 treatment did not affect tumor weights (Figure S6a–d, Supporting Information). Further, flow cytometric analysis revealed that CD3^+^ T cells were significantly increased in liver tumors of MYC‐ON mice after DU101 or DU102 treatment (Figure S6e,f, Supporting Information) as well as after *Arf1* knockout (Figure S6g, Supporting Information). CD3^+^ T cells were also significantly elevated in Hepa1‐6 allografts with DU101 or DU102 treatment (Figure S6h, Supporting Information). These data demonstrated that DU101 and DU102 treatment promoted T‐cell infiltration into tumors.

We also reported that *Arf1* knockdown in tumor cells resulted in the release of DAMPs, including ATP^2^ (Figure S7a,b, Supporting Information). We found that the released ATP level was also significantly increased in tumor cells with DU101 or DU102 treatment or Arf1 knockdown, which further stimulated T cell activation through P2RX7 (Figure S7a,b, Supporting Information). By detecting the phosphorylation level of well‐established TCR downstream kinases including ZAP70, LAT, and ERK, we found that T cells were activated after culturing with medium from tumor cells treated with either DU101 or DU102 or with *Arf1* knockdown (Figure S7c,d, Supporting Information). Simply adding ATP into the culture medium could reappearance the phenotype of elevated phosphorylation level (Figure S7e, Supporting Information). Furthermore, T cell activation was blocked after adding apyrase to the medium, indicating that the released ATP from the Arf1‐ablated tumor cells mediated T cell activation (Figure S7f–j, Supporting Information). Treated T cells with P2RX7 inhibitor, OxATP, also reversed T cell activation phenotype (Figure S7k, Supporting Information). The ratio of CD44^+^ CD62L^+^ central memory (T_CM_) cells was higher in hepatocellular carcinoma (HCC) tumors from MYC‐ON mice as well as in colon tumors from CT‐26 allograft mice after DU101 or DU102 treatment in comparison with DMSO treatment (Figure S7l–o, Supporting Information). Together, our data suggested that the treatment with the new Arf1 inhibitors did not directly kill tumor cells rather than enhanced anti‐tumor immunity through promoting T cell tumor infiltration, T cell activation through releasing ATP and T cell differentiation toward a durable memory T cell population, consistent with our previously reported effects of genetic ablation of *Arf1*.

### 2.5 The New Arf1 Inhibitors Inflame the Tumor Microenvironment

**Figure 3:**
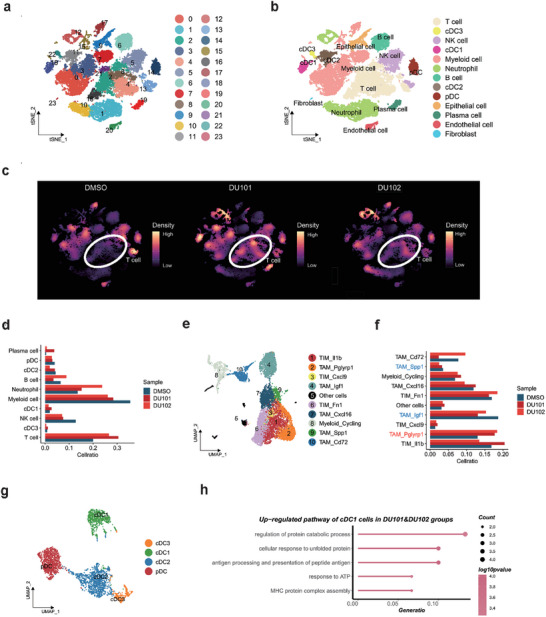
The new Arf1 inhibitors inflame the tumor microenvironment. a) Uniform manifold approximation and projection (UMAP) of immune and non‐immune cells in TME. b) Annotation of immune and non‐immune cells in TME. c) Cell density projection of immune and non‐immune cells in each treatment group. d) Bar plots showing the proportion of different types of immune cells in each treatment group. e) Annotation of Myeloid cells in TME based on selected cell markers. f) Bar plots showing the proportion of different subtypes of Myeloid cells in each treatment group. g) Annotation of DCs in TME based on selected cell markers. h) GO analysis of the up‐regulated genes of cDC1s in DU101 and DU102 treatment groups.

We next profiled a systematic analysis of the effects of DU101 and DU102 treatment on tumor‐infiltrating immune cells as well as cell‐cell interaction using single‐cell transcriptional profiling. To this, we performed single‐cell RNA sequencing (scRNA‐seq) with sorted CD45^+^ immune cells and CD45^−^ non‐immune cells from MYC‐ON liver tumors from DU101‐treated, DU102‐treated, and DMSO‐treated mice. We mixed sorted CD45^+^ immune cells and CD45^−^ non‐immune cells at the ratio of 10:1 for scRNA‐seq in each group, and obtained 35,468 cells in total after quality control and removing doublets. To find out the main components of TME, we integrated samples from different treatment groups, performed unsupervised clustering, and obtained 24 clusters of cells (**Figure** **3**a). We then annotated these cells using classical and well‐known markers and defined them as 13 cell types (Figure 3b; Figure S8a, Supporting Information). Comparing the cell proportion between different treatment groups, we found that the proportion of T cells significantly increased after DU101 or DU102 treatment in comparison with that of DMSO treatment, while the proportion of myeloid cells decreased (Figure 3c,d; Figure S8b, Supporting Information). These results suggested that the Arf1 inhibitors boosted tumor‐killing T cells.

We further examined myeloid cells, which accounted for the largest proportion of immune cells other than T cells. We obtained myeloid cells and performed unsupervised clustering and got 23 cell clusters (Figure S8c, Supporting Information). We then annotated myeloid cells based on their top feature genes and got 10 subtypes (Figure 3e; Figure S8d, Supporting Information). We observed an increase in the Pglyrp1 expressing TAMs and a decrease in the Spp1 and Igf1 expressing TAMs after DU101 and DU102 treatment (Figure 3f). The increased Pglyrp1 expressing TAMs exhibited pro‐inflammatory features and showed great ability in T cell recruitment and activation, as they highly express T cell chemokines Cxcl9 and Cxcl10, and type I IFN pathway genes Isg15 and Ifit2. In contrast, the decreased population of TAM_Spp1 and TAM_Igf1 showed immunosuppressive features, as they highly expressed genes associated with angiogenesis and immune regulatory factors, such as Arg1 and Spp1. To further evaluate the functions of myeloid cells, we collected previously reported macrophage M1 and M2 polarization gene lists^[^ ^25^ ^]^ and downloaded IFN production and response gene lists from the GSEA database. We found that the expression of M1 polarization genes, type‐I, and type‐II IFN production and response pathways were up‐regulated after DU101 and DU102 treatment, while M2 polarization genes were down‐regulated after DU101 and DU102 treatment (Figure S8e,f, Supporting Information). At last, we performed GO analysis on DEGs of myeloid cells after DU101 and DU102 treatment and found that pathways related to defense response, cytokine signaling pathway, and IFNβ pathway were enriched (Figure S8g, Supporting Information).

DCs play a crucial part in antigen‐presenting and innate immune response. We performed unsupervised clustering and annotated the 19 clusters of cells as cDC1, cDC2, cDC3, and pDC (Figure 3g; Figure S8h, Supporting Information). Because cDC1s are the major type of cells that mainly present antigen to CD8 T cells, we performed GO analysis on the up‐regulated DEGs of cDC1s after DU101 and DU102 treatment, and observed enrichment in antigen‐presenting pathway, suggesting an enhanced antigen presentation from cDC1s to CD8 T cells (Figure 3h). Furthermore, we selected several genes related to DC activation, antigen presentation, and IFN signaling pathway and observed their up‐regulation in different subtypes of DCs (Figure S8i, Supporting Information). These results demonstrated that myeloid cells were polarized into M1 pro‐inflammatory types and DCs were activated with enhanced antigen‐presenting ability after DU101 and DU102, the Arf1 inhibitors programmed myeloid subsets into a proinflammatory phenotype that is characteristic of an inflamed tumor microenvironment (TME).

The above data together demonstrated that the Arf1 inhibitors induced infiltration of anti‐tumor M1 macrophages, activated dendritic cells (DC), and reduced infiltration of tumor‐promoting M2 macrophages. Thus, the Arf1 inhibitors promoted proinflammatory remodeling of the TME to support anti‐tumor immunity.

### 2.6 The New Arf1 Inhibitors Reprogrammed T Cells with Superior Anti‐Tumor Activity

**Figure 4:**
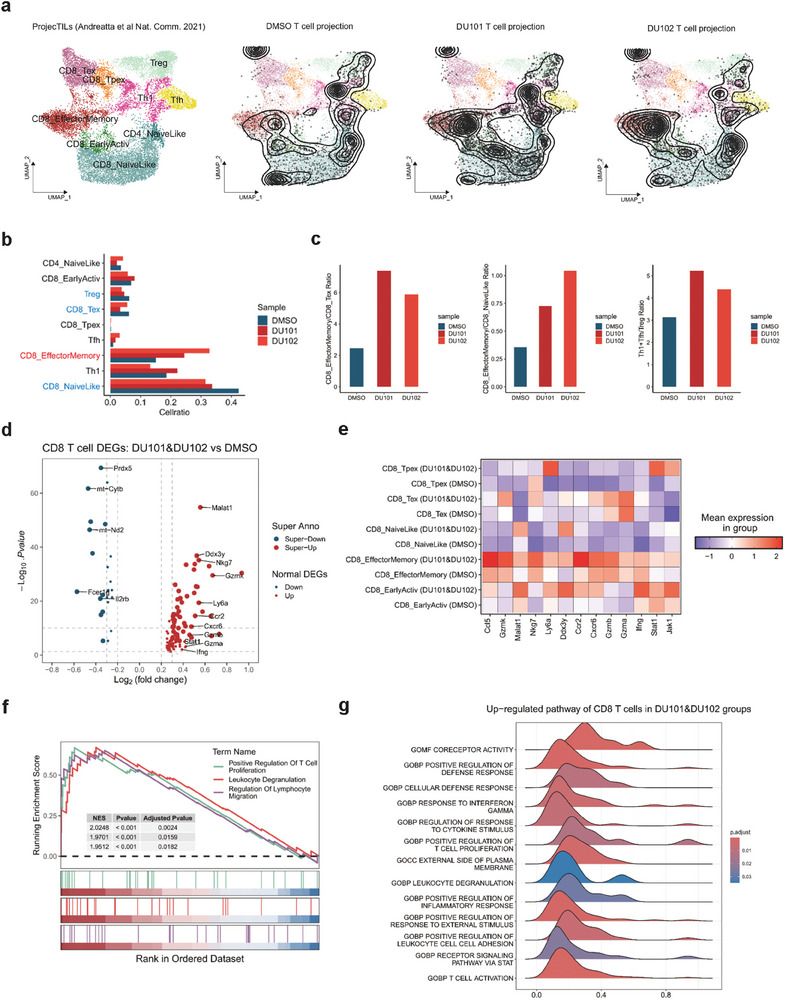
Treatment with the new Arf1 inhibitors induced T cell activation and blocked T cell exhaustion. a) Projection of T cells into reference T cell atlas by ProjecTILs. From left to right: Standard annotation of T cell subtypes in reference T cell atlas, projection of T cells in DMSO treatment group, DU101 treatment group, and DU102 treatment group. The contour lines show the cell density of projected T cells. b) Bar plots showing the proportion of different subtypes of T cells in each treatment group. c) The ratio of the proportion between different subtypes of T cells in each treatment group. Left: ratio of CD8_T_EffectorMemory cells versus CD8_Tex cells. Middle: ratio of CD8_T_EffectorMemory cells versus CD8_NaiveLike cells. Right: ratio of Th1 and Tfh cells versus Treg cells. d) Volcano plot showing up‐regulated and down‐regulated genes of CD8 T cells in DU101 and DU102 treatment groups. Threshold for Super Anno: |avg_log2FC| > 0.3 and ‐Log10. *p* value ≥ 5. Threshold for Normal DEGs: 0.2 ≤ |avg_log2FC| ≤0.3 and *p* value < 0.05. e) Heatmap showing expression of selected genes in different T cell subtypes in each treatment group. f,g) GSEA analysis of up‐regulated genes of CD8 T cells in DU101 and DU102 treatment groups. f) Three most representative up‐regulated pathways of CD8 T cells in DU101 and DU102 treatment groups. g) Ridge plots showing other 13 up‐regulated pathways of CD8 T cells in DU101 and DU102 treatment groups.

To further investigate the changes in T cells after treatment of the new Arf1 inhibitors, we obtained the annotated T cells and projected them to a reference mouse T cell atlas using ProjecTILs (**Figure** **4**a; Figure S9a, Supporting Information). We found that the proportion of CD8 T cells with effector and memory‐like features (CD8_EffectorMemory) increased, while the CD8 T cells with exhaustion‐associated features (CD8_Tex) and Tregs decreased after DU101 or DU102 treatment (Figure 4b). There was also a distinct increase in the ratio of CD8_EffectorMemory T cells to CD8_Tex cells, the ratio of CD8_EffectorMemory T cells to CD8_NaiveLike cells, and the ratio of the sum of Th1 cells and Tfh cells to Tregs (Figure 4c), suggesting that the T cells were more activated and had better tumor killing ability in addition to increased overall infiltration. Next, we analyzed the differentially expressed genes in CD8 T cells and found that there was a significant increase in the cytotoxicity‐related genes such as Gzma, Gzmb, Gzmk, and Nkg7, the T cell activation‐related genes Stat1 and Ifng, and the T cell chemotaxis related gene Cxcr6 (Figure 4d). We found that the cytotoxicity‐related genes and T cell activation‐related genes were up‐regulated most in CD8_EarlyActiv and CD8_EffectorMemory T cells (Figure 4e). After performing GSEA pathway enrichment analysis on the up‐regulated DEGs in DU101 and DU102 treatment groups, we found a significant enrichment in T cell proliferation, leukocyte cytotoxicity, lymphocyte migration, response to IFNγ, JAK‐STAT pathway activation, and so on (Figure 4f,g). Thus, DU101 and DU102 treatments reprogrammed CD8^+^ T cells into more activated T cells with elevated effector function, reduced exhaustion, increased proliferation, and stronger cytotoxic activity.

### 2.7 The New Arf1 Inhibitors Promote a Population of PD‐1^+^ TCF1^+^ CD8^+^ CD4^+^ Stem‐Like T Cells in Liver Tumors

**Figure 5:**
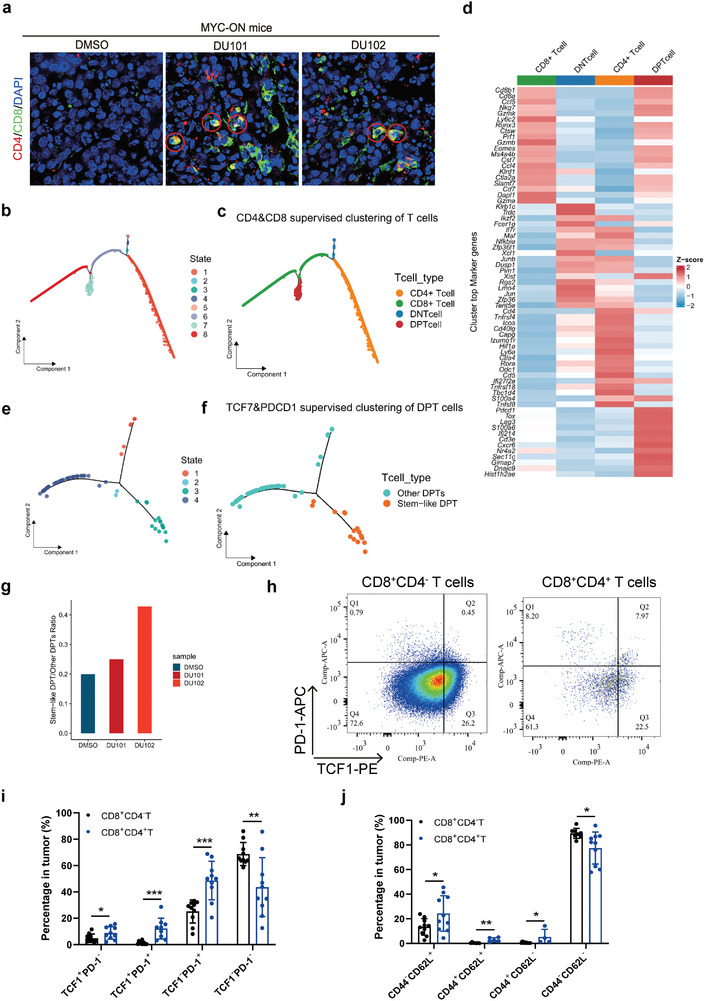
The new Arf1 inhibitors promote a population of PD‐1+TCF1+CD8+ CD4+ stem‐like T cells in liver tumors. Immunofluorescence staining (IF) of liver tumors from MYC‐ON mice after treatment with DMSO, DU101 or DU102. Red, CD4; Green, CD8; DAPI, Blue. White dotted circles outlined the CD8^+^ CD4^+^ T cells. Scale bar, 50 µm. (n = 3 mice). Data are shown as mean ± SEM. Student's *t*‐test. *p < 0.05, **p < 0.01, ***p < 0.001; n.s., not significant. a) Supervised clustering of T cells according to the expression of Cd4, Cd8a, and Cd8b1. b) Annotation of T cell states based on their expression of Cd4, Cd8a, and Cd8b1. c) Heatmap showing the top feature genes of CD8 SPT, CD4 SPT, DPT, and DNT cells. d) Supervised clustering of DPT cells according to the expression of Tcf7 and Pdcd1. e)Annotation of DPT cell states based on their expression of Tcf7 and Pdcd1. f) The ratio of stem‐like DPT cells versus other DPT cells in each treatment group. g) FACS Analysis of PD‐1^+^ TCF1^+^ stem‐like T cells in tumor infiltrated CD8^+^ CD4^−^ T and CD8^+^ CD4^+^ T cells. h) Analysis of PD‐1^+^ TCF1^+^ stem‐like T cells in tumor‐infiltrated CD8^+^ CD4^−^ T and CD8^+^ CD4^+^ T cells. i) Analysis of CD62L^+^ CD44^+^ central memory T cells in tumor‐infiltrated CD8^+^ CD4^−^ T and CD8^+^ CD4^+^ T cells.

Interestingly, we noticed a subpopulation of T cells with special CD4 and CD8 double positive also significantly increased in the DU101 or DU102 treated group in our scRNA‐seq analysis. First, we confirmed the significant increase in the special CD4 and CD8 double‐positive cells (DPT cells) in MYC‐ON liver tumors after DU101 and DU102 treatment or after *Arf1* gene deletion but not in spleens by FACS analysis (Figure S9b–e, Supporting Information). Immunofluorescence tissue staining of tumors from MYC‐ON mice also verified that the DPT cells were significantly increased in tumors from DU101 or DU102‐treated mice in comparison with those from vehicle‐treated mice (**Figure** **5**a). We then perform a systematic analysis of the CD8^+^ CD4^+^ T cell subpopulation. To get a pure cluster of CD8^+^ CD4^+^ T cells, we re‐clustered all T cells using a supervised pseudotime‐ordered analysis only using Cd4, Cd8a, and Cd8b1. T cells were clustered into 2 states of CD8 single positive T (SPT) cells, 1 state of CD4 SPT cells, 1 state of CD8^+^ CD4^+^ T cells, and several states of CD8 CD4 double negative T (DNT) cells (Figure 5b–c; Figure S9f, Supporting Information). According to feature gene comparison, the DPT cells were more similar to CD8 SPT cells (Figure 5d; Figure S9g, Supporting Information). Specifically, DPT cells highly expressed cell cytotoxic genes (Nkg7, Gzmk, Prf1, Eomes), T cell chemokine receptor (Cxcr6), and stem‐like T cell marker (Tcf7) (Figure 5d; Figure S9h, Supporting Information). Worth noting, we found that the percentage of stem‐like T cells (PD‐1^+^ TCF1^+^) was robustly increased in DPT cells after DU101 or DU102 treatment (Figure 5e–g). Consistently, the percentage of PD‐1^+^ TCF1^+^ stem‐like T cells was higher in DPT cells than in CD8 SPT cells in CT26 allografts (Figure 5h–i). Additionally, we found that DPT cells exhibited a greater potential for central memory T cell differentiation in vivo than CD8 SPT cells (Figure 5j).

In summary, we found that treatment of the new Arf1 inhibitors DU101 and DU102 enriched a CD8CD4 DPT cell population. The DPT cells have higher positive percentage of the stem‐like T cell marker TCF1 and T cell exhaustion marker PD‐1 (PD‐1^+^ TCF1^+^ CD8^+^ CD4^+^) and also had synergetic expression of memory T cell markers (including Sell/CD62L, CCR7, CD27, CD28, and IL7R), as well as activated T cell markers (including CD44, CD69, EOMES, and GZMB). The PD‐1^+^TCF1^+^CD8^+^ CD4^+^ T cells, first reported here according to our knowledge, might comprise a novel class of super anti‐tumor T cells.

### 2.8 CD8^+^CD4^+^ T Cells Exhibited Higher Activity and Stronger Cytotoxic Function Than CD8^+^ CD4^−^ T Cells

**Figure 6:**
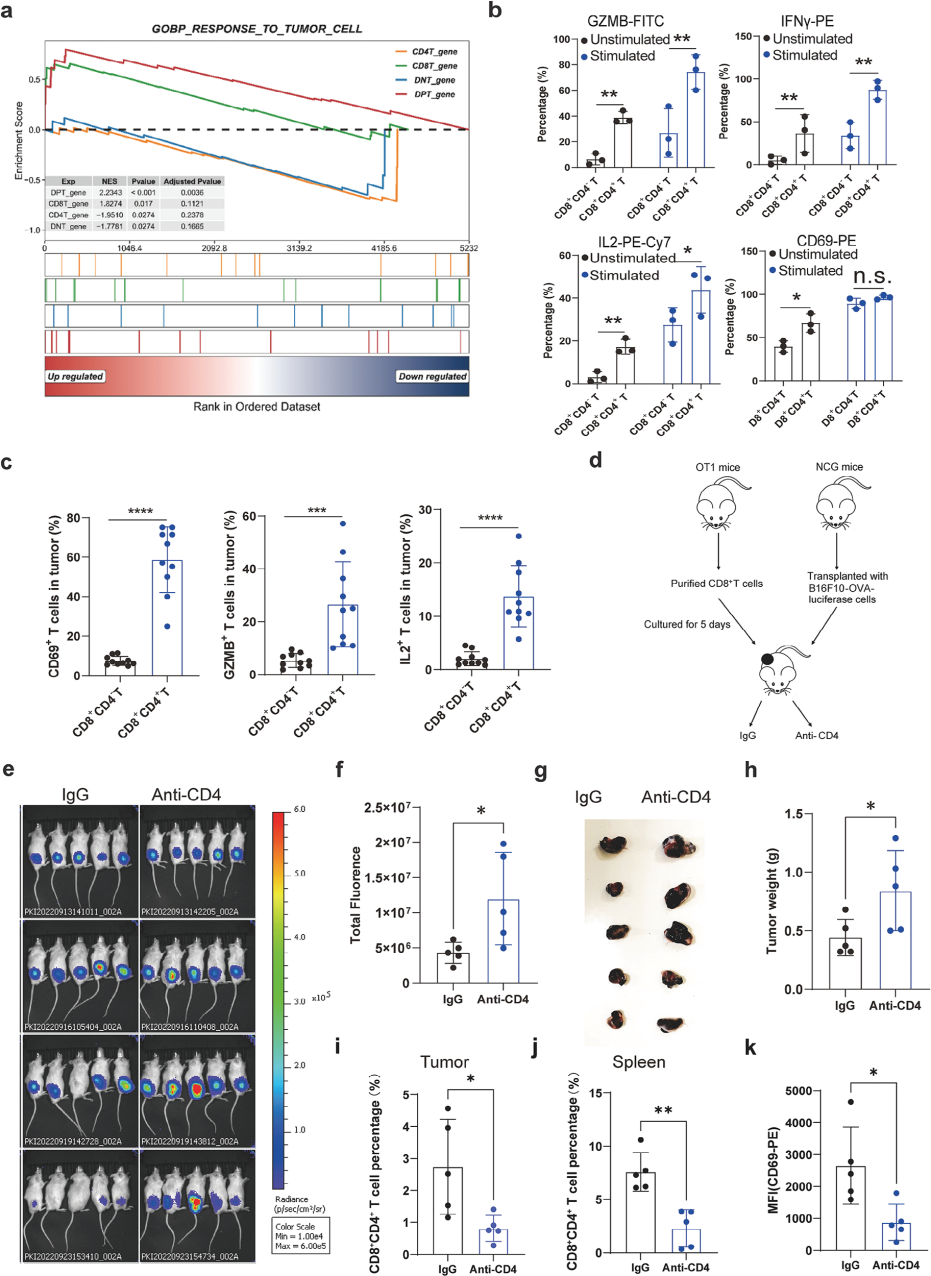
CD8^+^CD4^+^ T cells exhibited higher activity and stronger cytotoxic function than CD8^+^ CD4^−^ T cells. a) GSEA analysis of the top feature genes of CD8 SPT, CD4 SPT, DPT, and DNT cells. b) Quantification of CD69, GZMB, IFNγ and IL‐2 in CD8^+^ CD4^−^ or CD8^+^ CD4^+^ T cells after stimulation with anti‐CD3 and anti‐CD28 antibodies for 24 h. (n = 3). c) Quantification of the percentage of CD69^+^ T cells, GZMB^+^ T cells, and IL‐2^+^ T cells in CD8^+^ CD4^−^, or CD8^+^ CD4^+^ T cells from CT26 allograft tumors. (n = 10). d) The setup of adoptive T cell assay and anti‐CD4 treatment. e) Representative images of luciferase‐labeled tumors fluorescence in the two treatment groups of B16‐F10 allografts. (n = 5). f) Quantification of the total tumor fluorescence at Day 10. g) Representative tumor images of B16‐F10‐OVA‐Luciferase allografts after treatment with IgG or anti‐CD4. (n = 5). h) Tumor weights of B16‐F10‐OVA‐Luciferase allografts with IgG or anti‐CD4 treatment. (n = 5). i) Quantification of the percentage of CD8^+^ CD4^+^ T cells in tumor. (j) Quantification of the percentage of CD8^+^ CD4^+^ T cells in the spleen. k) Quantification of the mean fluorescence of CD69^+^ T cells in the tumor. Data are shown as mean ± SEM. Student's *t*‐test. *p < 0.05, **p < 0.01, ***p < 0.001, ****p < 0.0001; n.s., not significant.

We further performed GSEA analysis on the feature genes of these four (CD8 SPT, CD4 SPT, DPT, and DNT) T cell types, and found that DPT cells had superior characteristics in response to tumor cells, MHC protein binding, αβ T cell activation, and T cell selection, suggesting that DPT cells were more activated and probably had stronger anti‐tumor ability (**Figure** **6**a; Figure S10a, Supporting Information). To verify the activity of the DPT cells, we compared the activation marker expression level and cytokine expression levels of DPT cells with the CD8 SPT cells. The DPT cells displayed higher expression of CD69, GZMB, and IL‐2 than the SPT cells with or without anti‐TCR stimulation both in vitro (Figure 6b; Figure S10b, Supporting Information) and in CT26 allografts (Figure 6c). Though the percentage of CD69 showed no difference between DPT cells and CD8 SPT cells with anti‐TCR stimulation in vitro, the mean fluorescence is higher in DPT cells upon anti‐TCR activation (Figure S10b, Supporting Information). These data together suggested that DPT cells are more active potent cytotoxic T cells than CD8 SPT cells.

To characterize the role of DPT cells in T cell‐mediated anti‐tumor immunity in vivo, we transplanted CD8^+^ T cells purified from OT1 mice into immunodeficient mice with B16F10‐OVA tumors and then eliminated DPT cells by anti‐CD4 treatment (Figure 6d). We found that anti‐CD4 treatment significantly reduced the tumor‐killing function of the T cells, and tumor regression was slower in mice treated with anti‐CD4 than in mice treated with IgG (Figure 6e,f). The tumor size and weight were also larger in mice with anti‐CD4 treatment (Figure 6g–h). Accordingly, the DPT percentage was decreased both in the spleen and tumor in mice with anti‐CD4 treatment in comparison with IgG treatment (Figure 6i,j; Figure S10c–d, Supporting Information). Furthermore, the activation level of tumor‐infiltrated T cells, evaluated by the CD69 expression level, was decreased in mice with anti‐CD4 treatment in comparison with IgG treatment (Figure 6k; Figure S10e, Supporting Information).

### 2.9 The Stem‐Like DPT Cells Exist in Human Liver Tumors and Correlate with Better Disease‐Free Survival (DFS)

**Figure 7:**
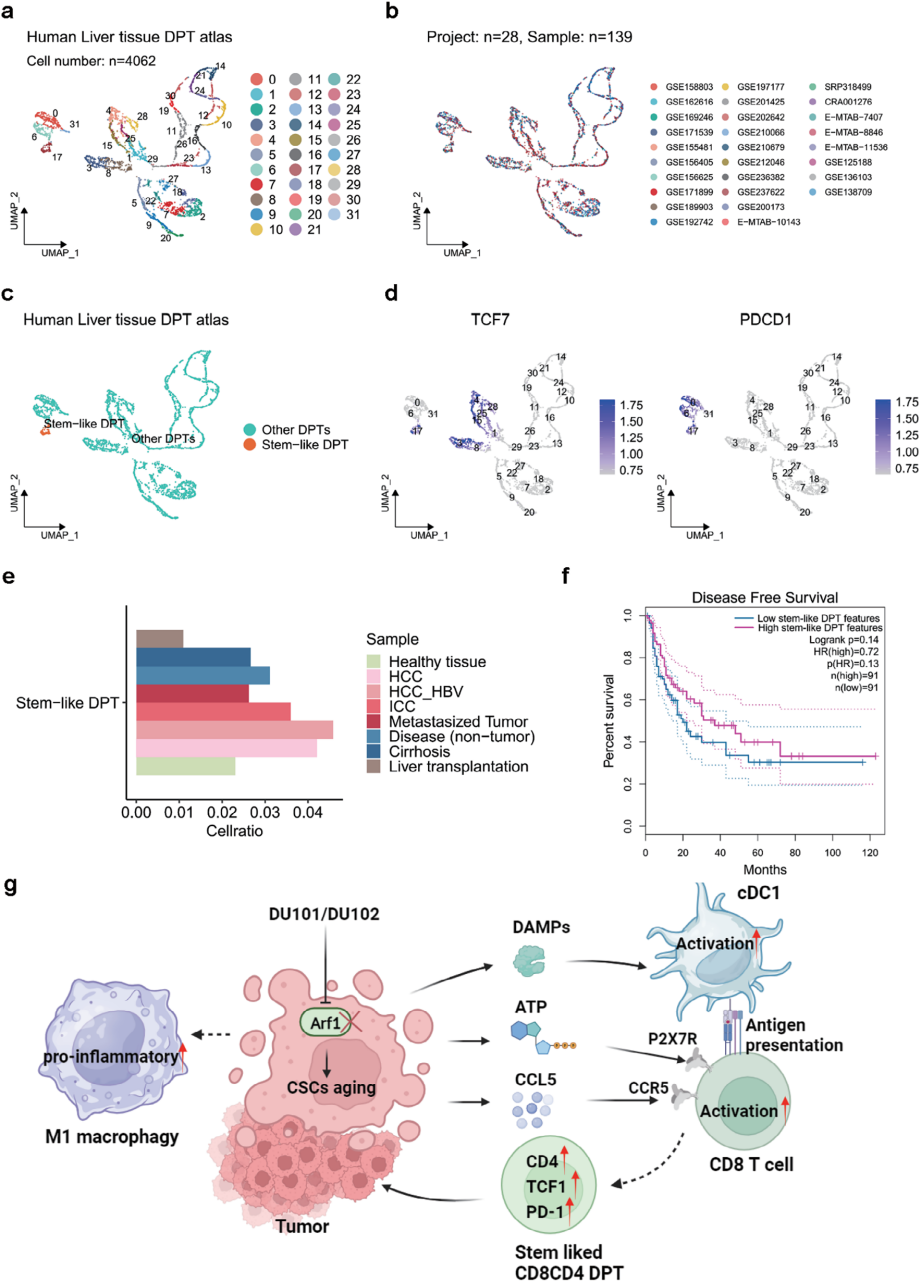
Construction of human liver tissue DPT cell atlas. a) Supervised clustering of human liver DPT atlas based on the expression level of CD4, CD8A, CD8B, TCF7, and PDCD1. b) Projects involved in the human liver DPT atlas. c) Annotation of DPT subtypes based on the expression of TCF7 and PDCD1 showed in (d). d) Feature plots showing the gene expression of TCF7 and PDCD1 in human liver DPT atlas. e) Bar plots showing the proportion of stem‐like DPTs in each type of patient sample. f) Kaplan–Meier plots showing the association of the expression of feature genes of stem‐like DPT cells in LIHC patients with a prognosis of disease‐free survival (DFS). g) Model of targeting Arf1 in tumor cells to induce systemic antitumor immunity.

We next tried to reveal whether this kind of DTP cells also existed in liver tumor patient samples. We utilized the deeply integrated human single‐cell omics data (DISCO), which was a public dataset of human scRNA‐seq data. We downloaded the liver tissue scRNA‐seq data from DISCO, filtered low‐quality cells, removed doublets, and isolated the potential DPT cells based on the positive expression of CD3, CD4, and CD8. We then constructed the first human liver tissue DPT cell atlas with 4062 high‐quality DPT cells from 28 projects and 139 samples. The supervised clustering was performed based on the expression of CD4, CD8A, CD8B, TCF7, PDCD1, and divided total DPT cells into 32 clusters (**Figure** **7**a,b). The DPT cells in cluster 17 expressed both TCF7 and PDCD1, which were identified as stem‐like DPT cells (Figure 7c,d). We clustered the samples and divided them into eight groups, and found that the stem‐like DPT cells had higher infiltration in tumor samples than normal tissues, which might be a result of tumor antigen stimulation (Figure 7e). We used the expression top ten feature genes of the stem‐like DPT cells together with CD3D, CD3E, CD3G, CD4, CD8A, and CD8B as gene signatures to analyze its association in human liver tumors with LIHC patients' prognosis. We find patients with higher expression of the stem‐like DPT gene signature tended to have better disease‐free survival (DFS) (Figure 7f), suggesting that the stem‐like DPT cells in human liver tumors could also mediate anti‐tumor immune response.

## 3 Discussion

### 3.1 A Potential Novel Dual Anticancer Therapy

In this study, we conducted a robust genetic screen in a Drosophila stem cell tumor system, testing over 100 modified chemicals from Exo2. This effort led to the identification of DU101 and DU102 as two potent small‐molecule inhibitors of Arf1 for cancer therapy. In both mouse models of cancer and pre‐clinical PDX tumor models, treatment with DU102 resulted in significant anti‐tumor immunity. We demonstrated that DU102 induces aging in cancer stem cells and triggers the release of various factors that remodel the tumor immune microenvironment. Consequently, it leads to the infiltration of anti‐tumor M1 macrophages, activated dendritic cells (DC) with enhanced antigen‐presenting ability, and CD8 effector T cells. Moreover, DU102 reduces the infiltration of tumor‐promoting M2 macrophages and exhausted CD8 T cells. Additionally, DU102 enhances T cell cytotoxicity and promotes the transformation of T cells to CD8CD4 double‐positive T (DPT) cells (Figure 7g).

### 3.2 The Effect of DU101 and DU102 May Target Tumor Cells with Stem Cell Features

The incidence of CSCs is generally less than 1% in most tumors. The question at hand is how the new Arf1 inhibitors in a small number of CSC cells could potentially trigger a robust systemic anti‐tumor response. In a recent study, it was discovered that pyroptosis of fewer than 15% of tumor cells was adequate to eradicate the entire grafted 4T1 mammary tumor.^[^ ^26^ ^]^ However, it's uncertain whether the new Arf1 inhibitors specifically target cancer stem cells (CSCs) to induce systemic anti‐tumor immunity due to the ongoing debate about CSC markers and their identity. The observed effects of DU101 and DU102 may have originated from CSC stress or cellular organelle aging crisis, which could have led to the release of danger signals or factors. These could have triggered the activation of cytotoxic CD8+ T cells, resulting in the elimination of CSCs and a shift from an immunosuppressive tumor microenvironment (TME) to a favorable immunogenic state. Additionally, the release of cancer‐associated antigens (CAAs) by the initial dying cells could have reactivated immune cells in the TME, initiating a new wave of immune responses and enhancing their magnitude with each subsequent round.^[^ ^26^ ^]^

### 3.3 The Novel Stem‐Like Double‐Positive T Cells Displayed Robust Anti‐Tumor Functionality

Previously, the focus has shifted towards stem cell‐like T cells as a result of their notable self‐renewal capacity, the ability for multi‐differentiation, and enduring effector function in the realm of adoptive therapy to combat tumors.^[^ ^27^ ^]^ TCF1+ PD‐1+ CD8+ stem‐like T cells have been identified as maintaining enhanced responses to immune checkpoint inhibitors (ICBs) and adoptive cell transfer (ACT) in therapeutic animal models and human subjects with tumors.^[^ ^28^, ^29^, ^30^, ^31^, ^32^, ^33^, ^34^ ^]^ Based on compelling evidence, it is clear that both CD8+ and CD4+ T cells are essential components in the body's anti‐tumor immune response.^[^ ^35^ ^]^ The DPT cells are known to potentially have additive effects. Nevertheless, recent research has indicated the presence of these cells in various human tumors, particularly in the leading‐edge area, which is situated adjacent to the malignant‐benign boundary of human hepatocellular carcinoma (HCC).^[^ ^36^, ^37^ ^]^ Human hepatocellular carcinoma (HCC) DPT cells demonstrate a high expression of PD‐1 and exhibit an activated phenotype along with robust functionality. Survival analysis indicates that HCC patients with a higher number of DPT cells and PD‐1+ DPT cells experience significantly improved overall survival and recurrence‐free survival rates,^[^ ^37^ ^]^ suggesting that the DPT cells are superior anti‐tumor T cells.

Studies pertaining to the lineage of DPT cells have yielded inconclusive findings. In the context of tumor development, the introduction of CD8+ T cells into B16 mice revealed distinct differentiation and functionalities of the DPT cells, emphasizing the adaptive nature of lineage commitment mechanisms within the peripheral environment.^[^ ^36^ ^]^ The TCR analysis of tumor‐infiltrated T cells in HCC patient samples indicated that the DPT cells likely originated from the same lineage as CD8 SPT cells, suggesting a probable transformation of DPT cells from intra‐tumoral SPT cells.^[^ ^37^ ^]^ In our study, we conducted a comparison of cell markers and differentially expressed genes. Our findings indicate that DPT cells demonstrate a gene expression profile more closely related to cells from CD8 SPT than CD4 SPT. Furthermore, our supervised pseudo‐time and cell trajectory analysis revealed a stronger resemblance in expression patterns between DPT cells and CCD8 SPT than with CCD4 SPT cells. This suggests a flexible differentiation between DPT cells and CD8 SPT and offers a potential strategy for enhancing DPT cells using small molecular chemicals. Given the low percentage of DPT cells, it is imperative to explore strategies for promoting DPT differentiation for potential clinical translation. Further analysis into the regulation of differentiation between DPT cells and CD8 SPT cells, along with careful consideration of their inclusion during the sequencing and analysis of TILs from patient samples, is warranted.

In conclusion, the newly developed Arf1 inhibitors in this study show promise as a novel strategy for cancer immunotherapy. With their strong pharmacology and positive safety profile in preclinical toxicology studies, a new and improved derivative (AOB01) of DU101 and DU102 is currently undergoing evaluation in Investigational New Drug (IND) studies, both as a monotherapy and in combination with anti‐PD‐1 for solid tumors. As far as we are aware, this new Arf1 inhibitor is the pioneering drug that activates a dual anticancer therapy by promoting cancer stem cell aging and anti‐tumor immunity and may lay the groundwork for additional therapeutics targeting this dual process.

## 4 Experimental Section

### Experimental Model and Subject Details—Mice

For the study, MYC‐overexpressing liver cancer mice were generated by breeding B6.Cg‐Tg(Cebpb‐Tta)5Bjd/J (JAX:003563) and FVB/N‐Tg(tetO‐MYC)36aBop/J (JAX:019376) mice, both obtained from The Jackson Laboratory. NOD/ShiLtJGpt‐Prkdc^em26Cd52^Il2rg^em26Cd22^Hr^em1Cin8936^/Gpt mice (Cat# T003257) were also purchased and CD34^+^ HSC‐NOD/ShiLtJGpt‐Prkdc^em26Cd52^Il2rg^em26Cd22^/Gpt mice (Cat# T037620) were humanized from GemPharmatech in Nanjing, China. Additionally, OT1 mice were kindly provided by Prof. Yu Xiaofei of Fudan University. Age‐matched male and female mice were used for all experiments, with 6‐week‐old mice being the starting point for all experiments. Furthermore, PDX transplantation was performed using 12‐week‐old humanized mice. All animals were housed in specific pathogen‐free facilities, and all procedures were conducted in compliance with the Animal Care and Use Committee (ACUC) at Fudan University.

### Experimental Model and Subject Details—Cells

Mouse colorectal carcinoma CT26 cells (Cat# CRL‐2638) derived from BALB/c mice, mouse melanoma B16‐F10 cells (Cat# CRL‐6475) derived from C57BL/6J mice, mouse stage IV breast cancer 4T1 cells (Cat# CRL‐2539) derived from BALB/c mice, human embryonic kidney HEK 293T/17 cells (Cat# CRL‐11268), mouse hepatoma Hepa1‐6 cells (Cat# CRL‐1830) derived from C57BL/6J mice, and mouse Lewis lung carcinoma LLC1 cells (Cat# CRL‐1642) derived from C57BL/6J mice were all obtained from the American Tissue Culture Collection (ATCC). B16‐F10 cells expressing OVA antigen were kindly provided by Dr. Gaofeng Fan of Shanghaitech University. CT26 cells and 4T1 cells were cultured in RPMI 1640 (SIGMA, Lot# RNBK0103) containing 10% fetal bovine serum (FBS) (HyClone, Cat# SH30396.03) and 100 units mL^−1^ penicillin/streptomycin (Gibco, Cat# 15140122). B16‐F10 cells, HEK 293T/17 cells, Hepa1‐6 cells, and LLC1 cells were grown in DMEM (Sigma, Lot# RNBK3466) supplemented with 10% FBS and 100 units mL^−1^ penicillin/streptomycin. All cells were cultivated in a humidified atmosphere containing 5% CO_2_ at 37 °C.

Primary cultures of mouse T cells from the spleen were performed as previously described.^[^ ^27^ ^]^ Male and female mice at 8–12 weeks old were euthanized by CO_2_, and the spleen was collected and dissected by grinding. CD8^+^ T cells were separated using the EasySepTM Mouse CD8 T Cell isolation Kit (Stemcell, cat. No. 19853). T cells were re‐suspended and activated in anti‐CD3 and anti‐CD28 mAbs‐coated plates (invivomab) for 48 h. Activated T cells were maintained with IL‐2 (10 ng mL^−1^) and 2‐mercaptoethanol and cultured with a fresh complete medium.

### Genomic Mouse Models

In the context of the liver tumor model in mice, as previously documented in the scientific literature under PubMed ID: 31924786, Cebpb‐tTA/tet‐o‐MYC/Axin2‐CreER/Arf1f/f mice was employed to specifically ablate the Arf1 gene in Axin2+ liver cancer stem cells using Axin2‐CreER in the MYC‐ON mice. The alimentation provided in both the mating cage and the wean cages consisted of a Grain‐Based Rodent Diet containing 200 mg kg^−1^ doxycycline (Bio‐Serv, Cat# 14‐727‐450). Tumorigenesis was initiated in 6‐week‐old mice through a transition to a normal diet. In Cebpb‐Tta/tet‐o‐MYC/Axin2‐CreER/Arf1f/f mice, the excision of the Arf1 gene from the stem cells was achieved via intraperitoneal injection of 10 mg kg^−1^ tamoxifen (Sigma–Aldrich, Cat# T5648) daily for a period of five consecutive days. The euthanasia of the mice was performed subsequent to the manifestation of tumors, at 10–12 weeks.

### Engrafted Tumor Models

For grafts of mouse tumor cells in BALB/c or C57BL/6J background mice, littermate mice were separated into groups, and 5 × 10^5^ CT26 cells, 5 × 10^5^ 4T1 cells, 5 × 10^5^ B16‐F10 cells or 5 × 10^5^ Hepa1‐6 cells were injected subcutaneously (s.c.) into the mouse's left or right side. DU101 or DU102 (5 mg k^−1^g or 1% DMSO) was given to mice by gavage daily. Tumor volume was measured every 2–3 days by a digital caliper (ULINE, Cat# H‐7352), and the tumor volume was calculated using the formula ½ × longitudinal diameter (length) × the greatest transverse diameter (width).^[^ ^2^ ^]^ Mice were euthanized when the tumor volume reached 2000 mm^3^.

### Patient‐Derived Xenograft (PDX) Implantation

The liver tumor patient‐derived xenograft (PDX) experiments were conducted in strict accordance with ethical guidelines, adhering to the principles outlined in the Declaration of Helsinki. Informed consent was obtained from all patients or their legal guardians. The primary liver tumor specimens originated from patients with liver cancer who had undergone clinical surgery at Zhong Shan Hospital, Fudan University in Shanghai, China. Following surgical resection, the liver tumors were sectioned into 1 mm^3^ fragments and immersed in sterile 1xPBS. These minute tumor fragments were subsequently subcutaneously transplanted into anesthetized 11‐week‐old humanized mice. The mice were housed in controlled, pathogen‐free environments with a 12‐h dark/light cycle, and daily monitoring of tumor growth was performed.

### Phenotypic Screen for Small Molecules That Kill Stem Cell Tumors in *Drosophila*

As previously demonstrated that knockdowns of the COPI/Arf1‐lipolysis pathway selectively killed Ras^V12^‐transformed stem cell tumors in *Drosophila* Malpighian tubules (MT, fly kidney) through necrosis but spared differentiated cells. Feeding flies with the existing Arf1 inhibitors selectively killed tumorigenic stem cells but not normal stem cells.^[^ ^3^ ^]^ Using the *Drosophila* stem cell tumor system, a phenotypic assay was developed using fluorescent confocal microscopy and screened new compounds that killed Ras^V12^‐induced stem cell tumors.

The screening was conducted in 96‐well plates, modified from a published screening procedure successfully conducted in adult *Drosophila*.^[^ ^28^ ^]^ In brief, i) after clonal induction (ACI) using the positively marked mosaic lineage (PMML) labeling technique^[^ ^29^ ^]^ in adult *Drosophila*, flies with Ras^V12^‐transformed renal and nephric stem cells (RNSCs) were cultured at 29 °C for 4 days to allow for tumor growth; ii) fly food was boiled, cooled to 37 °C, aliquoted into plates at 100 µL well^−1^; (iii) drugs were added at 0.5 µL well^−1^ and mixed by pipetting up and down five times, resulting in a final compound concentration of ≈50 µM; and iv) after food solidification, flies with Ras^V12^‐transformed RNSCs were added to the wells (three females per well) and cultured for 3 days at 29 °C. The flies were dissected after incubation and viewed under a dissecting microscope, and a digital camera was used to capture images of tumors using white light and GFP fluorescence. The data were analyzed using MetaMorph (Molecular Devices) to manually set a pixel intensity threshold that included the GFP‐labeled tumors but not background fluorescence. The data for each compound were stored in a Microsoft Access database. Drugs that reduced GFP levels by 50% or greater were retested in three wells with nine flies. Drugs that reduced GFP levels by 50% or more in all three replicates relative to DMSO were scored as hits.

The classic Arf1 inhibitor brefeldin A (BFA) and the small molecule 4‐hydroxy‐3‐methoxybenzaldehyde (5,6,7,8‐tetrahydro[1]benzothieno‐[2,3‐d]pyrimidin‐4‐yl) hydrazone (Exo2) stimulate biological changes that are virtually indistinguishable. Exo2 is much more amenable to chemical modification.^[^ ^30^ ^]^ A range of existing and newly synthesized Exo2 analogs were screened and it was found that DU101 and DU102 were effective and had relatively low toxicity (Patent No. PCT/CN2021/110 373).

### Generation of Cell Lines

The detailed sequences of the shRNA oligonucleotides are listed in Table S1. The double oligonucleotides were annealed at 95 °C for 20 min and cloned into the pLKO.1‐Puro lentivirus vector. After sequencing, the pLKO.1 plasmids containing different shRNAs were obtained. Lentiviruses carrying the pLKO.1 plasmids were then produced by co‐transfecting HEK293T cells with two helper plasmids: pVSVG and pMD2.G. The lentiviral particles were harvested from the medium after 48 h of co‐transfection. The cell medium was passed through a 0.22‐µm filter, and the lentivirus was collected into Eppendorf tubes and kept at −20 °C for short‐term storage or −80 °C for long‐term storage. The cells were infected and selected with 5 µg mL^−1^ puromycin treatment (ThermoFisher, Cat# A1113803) for 2 days. Stable cell lines were cultured in 1.0 µg mL^−1^ puromycin.

### Immunohistochemistry (IHC) and Immunofluorescence (IF) Analyses

Mouse tissue and tumors were dissected and fixed in 4% paraformaldehyde at 4 °C overnight after mice were euthanized by CO_2_ for 20 min. Tumors were dehydrated by gradient alcohols or 30% sucrose and then embedded in paraffin wax or OCT (Tissue‐Tek; Sakura Finetek USA, Cat# 4583). 5um paraffin sections were generated by a Leica RM2125 Microtome. 10um frozen sections were generated by a Leica CM1950. Paraffin sections were dewaxed with xylene and hydrated with gradient alcohol. Antigens were retrieved by sodium citrate solution (Solarbio, Cat# C1032) in a 100 °C cooker for 25 min. The sections were washed in 1xPBS three times for 5 min each and blocked in 3% FBS for 1 h at room temperature. Then, the sections were incubated with primary antibodies at 4 °C overnight. The following day, sections were washed in 1xPBS three times for 5 min each. For IHC, the paraffin sections were incubated with secondary antibodies for 1 h at room temperature and then stained with a DAB horseradish peroxidase color development kit (Beyotime, Cat# P0202). For IF staining, the frozen sections that had been incubated with primary antibody were then stained with fluorescent secondary antibody at room temperature and protected from light for 1 h. The sections were examined with an Olympus inverted fluorescence microscope (NEW IX73) or a Zeiss (LSM710) confocal microscope.

### T Cell Depletion

Mice were treated with 200 µg per mouse of rat anti‐CD4 (clone GK1.5, BioXcell, Cat#BP0003‐1) or 200 µg per mouse of rat IgG (BioXcell, Cat#BP0090) isotype control antibodies. All antibodies were diluted in PBS and i.p. injected into mice for 3 consecutive days per week, for two consecutive weeks.

### T Cell Isolation and Culture

To isolate spleen cells, a mouse was euthanized by CO_2_ for 20 min, and the spleen was harvested, minced into small pieces, and placed in RPMI 1640 medium. The minced spleen was dissociated by pipetting up and down with a 5‐mL syringe plunger. The cells were then passed through a 40‐µm strainer (JET BIOFIL, Cat# CSS013040), followed by the lysis of red blood cells. The isolated cells were washed twice in cell staining buffer (0.5% BSA in 1 × PBS). CD8‐positive T cells were purified with the EasySep Mouse CD8+ T Cell Isolation Kit (Stemcell, Cat# 19853). After centrifugation, T cells were resuspended in RPMI 1640 medium, 10% FBS, 100 units mL^−1^ penicillin/streptomycin, 4 mM L‐glutamine (Gibco, Cat#25030081), 1 mM sodium pyruvate (Gibco, Cat# 11360070), 50 µM 2‐mercaptoethanol (Sigma–Aldrich, Cat#M3180), 25 mM HEPES (Gibco, Cat#15630130), and 1 × MEM nonessential amino acid solution (Gibco, Cat#11140050). T cell suspensions were placed in plates that had been incubated one day earlier with anti‐CD3 and anti‐CD28 antibodies at a final concentration of 3 µg mL^−1^. Two days later, recombinant mouse IL‐2 (Genescript, Cat# Z02764) at a final concentration of 10 ng mL^−1^ was added to the medium. Mouse T cells were cultured in a humidified atmosphere containing 5% CO_2_ at 37 °C.

### T Cell Activation Assay

Tumor cells were cultured with 10 µM DU101 or DU102 for 24 h and changed to normal medium for another 24 h. Then, the cell medium was collected and centrifuged for 3 min at 1,000 rpm and stored at 4 °C. To digest ATP, the cell medium was treated with 10 U mL^−1^ Apyrase (NEB, Cat# M0398) at 30 °C for 30 min. T cells (6 × 10^6^) were cultured with cell conditioned medium for 24 h. The T cells were then collected by centrifugation at 500 × g for 3 min, resuspended in 600 µL RPMI 1640 medium without FBS, and cultured at 37 °C for 60 min. The T cells were divided into 3 portions and stimulated with anti‐CD3 antibody for 30 s at 37 °C. Then, the T cells were treated with a secondary antibody (Thermo Fisher, Cat# A18891) containing sodium orthovanadate for the indicated time. The T cells were lysed with RIPA buffer and boiled at 95 °C for 20 min. The protein samples were centrifuged at 12 000 rpm for 10 min at 4 °C and subjected to western blotting.

### T Cell Adoptive Experiment

CD8^+^ T cells isolated from OT1 mice were treated with anti‐CD3 and anti‐CD28 for 2 days. 1 × 10^6^ B16‐F10‐OVA‐Luciferase cells were subcutaneously injected into NCG mice. After 4 days, 1 × 10^6^ CD8^+^T cells were adoptively transferred into tumor‐bearing mice via tail vein injection.

### Tumor‐Infiltrated Immune Cell Isolation

Mouse liver tumor‐infiltrated immune cells were isolated by mincing them into small pieces and dissociating them with a tumor dissociation kit (Miltenyi Biotec, Cat# 130‐096‐730). The cells were collected and passed through a 40‐µm strainer into a 50‐mL tube. The single‐cell suspension was centrifuged for 5 min at 2000 rpm at room temperature. The pellet was washed with RBC lysis buffer (Beyotime, Cat# C3702) for 3 min, and the cells were spun for 5 min at 2000 rpm at 4 °C and resuspended in RPMI medium to prepare the cells for staining for flow cytometry.

### Fluorescence‐Activated Cell Sorting (FACS)

The cells were suspended in a cell staining buffer (4abio, Cat# FXP005). DAPI (Sigma‒Aldrich, Cat# D9542) or a Zombie NIR Fixable kit (Biolegend, Cat# 423105) was used to identify live or dead cells. Cell surface staining was performed by adding appropriately conjugated fluorescent primary antibodies to the cells and incubating them at room temperature for 1 h in the dark. For intracellular staining, the cells were first fixed in 4% paraformaldehyde and permeabilized with 0.1% Triton X‐100 (Beyotime, Cat# ST795), and then intracellular staining was performed according to the corresponding protocols. After staining, suspended cells were analyzed by flow cytometry. Flow cytometry was performed using an LSR Fortessa (BD). Data were analyzed and presented with FlowJo_V10 software. The antibodies used are listed in Table  S2 (Supporting Information).

### Plasmids

The pET21b pVSVG, pMD2.G, and pLKO.1‐puro were kindly provided by Dr. Gaofeng Fan of Shanghaitech University. The other plasmids were generated in‐house; the detailed sequences are shown in Table S1 (Supporting Information). Briefly, all of the shRNAs were synthesized by Sangon, and the pLKO.1‐Puro plasmids were double digested by the AgeI‐HF (New England Biolabs, Cat# R3552S) and EcoRI‐HF (New England Biolabs, Cat#R3101S) enzymes. The shRNAs and vector were ligated by T4 DNA ligase (New England Biolabs, Cat# M0202S). Positive clones were sequenced by Sangon to verify that the ligation was correct.

### Western Blotting

Cell proteins were released by RIPA lysis buffer (0.1% SDS, 150 mM NaCl, 0.5% sodium deoxycholate, 1 mM EDTA, 1 mM EGTA, 1.0% Triton X‐100, and 50 mM Tris pH 8.0) containing 1 mM PMSF (Beyotime, Cat# ST505). The proteins were then centrifuged at 12 000 × g for 3 min at 4 °C. The supernatant protein concentration in the lysates was determined by a Protein Assay kit (Bio‐Rad, Cat# 5000006). All samples were adjusted to equal concentrations, 5x SDS loading buffer was added, and the sample was boiled at 100 °C for 15 min. Equal volumes of protein samples were loaded onto protein gels and separated by electrophoresis with an 80 V PowerPac Basic Power Supply (Bio‐Rad) for 100 min, then transferred to a nitrocellulose membrane (GE Healthcare, Cat# 10600014). The membrane was blocked with 5.0% skimmed milk powder for 1 h and then incubated with the appropriate primary antibody at 4 °C overnight with shaking. The next day, the membrane was washed with 1 x PBST three times for 5 min and incubated with the appropriate secondary HRP antibody in 5% milk for 1 h. The membrane was then developed with an Omni‐ECLFemto Light Chemiluminescence Kit (Epizyme, Cat# SQ201 L) and imaged with an imaging system (Tanon 5200).

### RNA Extraction and qRT‐PCR

For quantitative reverse transcription polymerase chain reaction, total RNA was extracted from the cells with a kit (Beyotime, Cat# R0011), and cDNA was synthesized by the Hifair V One‐Step RT‐gDNA Digestion SuperMix for qPCR Kit (Yeason, Cat# 11142ES60). The primers used for qRT‐PCR are listed in Table S1 (Supporting Information). The total cDNA was amplified by Hieff qPCR SYBR Green Master Mix (No Rox) (Yeason, Cat# 11201ES08) with a CFX96 Touch Real‐Time PCR Detection System (Bio‐Rad). The 18S gene was used as an internal control. The results were calculated as 2^−ΔCT^.

### Imaging of Grafted Tumors

Mouse melanoma B16F10‐OVA cells were transfected with pmeLUC plasmids and selected with 200 µg mL^−1^ G418 (Beyotime, Cat# ST082) for 20 days to generate a stable cell line. NCG mice were s.c. grafted with 1 × 10^6^ B16F10‐OVA‐LUC cells. D‐luciferin (100 mg kg^−1^) was injected into the mice i.p. 10 min before imaging. The mouse tumor sites were photographed with a Xenogen IVIS Spectrum at the animal facility of the Institute of Developmental Biology and Molecular Medicine of Fudan University (Shanghai, China). The in vivo bioluminescence of the tumor region was quantified using Living ImageH software.

### Extracellular ATP Test

Cells were cultured in RPMI164 medium overnight, and the cells were treated with 10 µM DU102 or DU101 the next day. After 48 h of treatment, the cell medium was collected and centrifuged at 4 °C at 1000 rpm for 10 min to clear dead cells. The supernatant was collected for the ATP test according to the manufacturer's instructions. Briefly, 10 µL of CCK‐8 solution was added to 100 µL of the sample. Then, the samples were incubated for 1 h at 37 °C. The absorbance was measured at 450 nm with a microplate reader.

### Protein Purification

To prepare Sec7 and Arf1 proteins, the gene fragment of Sec7 or Arf1 was ligated into vector pET‐21b (+). After validating the correct sequence, the recombinant plasmids were transformed into BL21(DE3) competent cells (Transgen, Cat# CD601). Isopropyl‐β‐D‐thiogalactoside was added to the bacteria at a final concentration of 1 mM when the OD600 reached 0.6 to induce protein expression. After incubating at 16 °C for 16 h, the bacteria were collected, and the His‐tagged proteins were extracted using a protein purification kit (Beyotime, Cat# P2229S) according to the manufacturer's instructions.

### Arf1 Activity Assay

Cells were cultured in RPMI 1640 overnight and transfected with the p23‐GGA3‐FLAG plasmid the next day. After 24 h, the cells were treated with GCA, BFA, DU101 or DU102. After 24 h of treatment, the cells were washed with 1X PBS and lysed with IP buffer (25 mM Tris‐HCl, pH 7.4), 150 mM NaCl, 1% NP‐40, 1 mM EDTA, 5% glycerol, and protease inhibitor). The cell lysates were centrifuged at 13 000 × g for 10 min, and the supernatant was transferred to a new 1.5 mL centrifuge tube. The protein concentration was detected by a BCA Protein Assay Kit (Beyotime, Cat# P0012). An equal amount of protein was used in the GGA3‐IP experiment. 500 µL supernatant was mixed with 10 µL of 0.5 M EDTA pH 8.0, 5 µL of 10 mM GTPγS, and 5 µL of 100 mM GDP and vortexed. The mixture was incubated at 30 °C for 15 min with constant agitation. To terminate the reaction, 32 µL of 1 M MgCl2 was added to the mixture and vortexed. Then, Flag‐tagged magnetic beads (Beyotime, Cat# P2181M) were added to the mixture and incubated at 4 °C overnight. After incubation, the magnetic beads were washed 3 times, resuspended in protein loading buffer, and examined by immunoblotting with anti‐Arf1 antibody (Proteintech, Cat# 10790‐1‐AP). Protein bands were visualized with an Omni‐ECL Femto Light Chemiluminescence Kit (Epizyme, Cat# SQ201 L) on a Bio‐Rad ChemiDoc Touch (America).

### Single‐Cell Sequencing

MYC‐ON mice treated with DU101, DU102, or DMSO for 6 weeks were euthanized. Liver tumors were minced into small pieces and dissociated with a mouse Tumor Dissociation Kit (Miltenyi Biotec, Cat# 130‐096‐730). The single‐cell suspension was centrifuged for 5 min at 2000 rpm at room temperature. This pellet was washed with RBC lysis buffer (Beyotime, Cat# C3702) for 3 min, and the cells were spun for 5 min at 2000 rpm at 4 °C. Then, the cells were stained with the fixable viability dye ZomBie NIR and anti‐mouse CD45‐PE antibody as well as anti‐mouse CD326 (Ep‐CAM)‐FITC, and sorted by BECKMAN COULTER MoFlo XDP. FACS sorted CD45+ cells and CD45‐ cells at the ratio of 10:1, and cells were sequenced by the 10x Genomics Chromium Single Cell platform. The barcoded libraries were prepared following the manufacturer's guidelines. Subsequently, 3′ mRNA libraries were created and subjected to sequencing on one NextSeq run and one NovaSeq SP run. Each sample yielded a count of over 315 million reads during sequencing. The sequencing was set up as a 28 cycles + 75 cycles non‐symmetric run on the NovaSeq. Demultiplexing was performed, allowing for one mismatch in the barcodes. More than 97.2% of bases in the unique molecular identifier (UMI) have Q30 or above.

### Quality Control of scRNA‐seq Data

Computational analyses were performed in R (v4.2.1) and Python (v3.10). For quality control, first SoupX^[^ ^38^ ^]^ was used to quantify and remove contamination caused by ambient mRNAs after Cellranger.^[^ ^39^ ^]^ Then Scrublet^[^ ^40^ ^]^ was used to detect doublets in single‐cell RNA‐seq data. Then a Seurat object was created using Seurat (v4.2.0)^[^ ^41^ ^]^ and only the cells predicted to be “FALSE” by Scrublet were retained. To exclude low‐quality cells and erythroid cells before hierarchical clustering, only the cells that met the following requirements were retained: i) 250 < nFeature_RNA < 6000, ii) percent.mt < 15, iii) percent.hb < 0.5. To exclude genes with extremely low expression, only the genes with more than 10 TRUE values were retained.

### Multiple Sample Integration and Unsupervised Clustering

For unsupervised clustering of all tumor‐resident cells, Seurat object was normalized using NormalizeData. 2000 highly variable genes were selected using FindVariableFeatures. Then the data were scaled in all genes using Scaledata and performed Principal Components Analysis (PCA) with RunPCA. Harmony^[^ ^42^ ^]^ was used to integrate three samples (DU101, DU102 and DMSO). FindNeighbors were performed on the top 40 principal components and RunTSNE or RunUMAP were used for data visualization.

### Supervised Clustering Analysis

Monocle 2^[^ ^43^ ^]^ was used to perform supervised clustering on T cells based on the expression of Cd4, Cd8a, and Cd8b1 to isolate DPT cells and supervised clustering on DPT cells was performed based on expression of Tcf7 and Pdcd1 to isolate stem‐like DPT cells. In detail, ordering_genes were set as Cd4, Cd8a, and Cd8b1, or Tcf7 and Pdcd1, and run setOrderingFilter with parameters as ordering_genes.

### Public scRNA‐seq Dataset Analysis and DPT Atlas Construction

DISCOtoolkit (https://github.com/JinmiaoChenLab/DISCOtoolkit) was used to filter and download data from DISCO dataset.^[^ ^44^ ^]^ The FilterDiscoMetadata function was used with the parameter tissue = liver and DownloadDiscoData was used to download the data. First low‐quality cells were excluded and only kept cells with “percent.mt” less than 15. Then T cells were isolated based on the non‐zero expression of CD3D CD3E or CD3G. Next, the doublets were removed using DoubletFinder.^[^ ^45^ ^]^ In the end, DPT cells were isolated from total T cells based on the non‐zero expression of CD4 and CD8 genes (CD8A or CD8B). The same procedures were followed as the mouse data analysis was done and unsupervised and supervised clustering was performed to construct the human liver tissue DPT cell atlas.

### Drug Affinity Responsive Target Stability (DARTS)

The drug affinity responsive target stability experiment was performed as previously reported^[^ ^31^, ^32^ ^]^ Briefly, Cell lysates from independent biological replicates were aliquoted in equivalent volumes containing 100 µg of protein and incubated for 30 min at 25 °C with or without DU101 or DU102. Proteinase K from *Tritirachium album* (Sigma–Aldrich, St. Louis, MO, USA) was added simultaneously to all samples at a proteinase K: substrate mass ratio of 1:100 and incubated at 25 °C for 30 min.

### Molecular Docking

Relevant targets were entered on the UniProt website (https://www.uniprot.org/) to search and download the human protein structure with a eutectic molecule (PDB ID: 1RE0) as the docking receptor, and then AutoDock Tools 1.5.6 software was used to dehydrate the protein, hydrogenate it, calculate the Gasteiger charge and save it as a PDBQT file. Small molecule compounds were downloaded from PubChem for the 2D structure (https://pubchem.ncbi.nlm.nih.gov/) with ChemBio3D energy minimization and AutoDock Tools 1.5.6 software to add atomic charge and distribution of atom types for the compound. The rotation center was selected, and all flexible keys were rotated by default and saved in PDBQT format as the docking ligand. With proteins as receptors and small molecules as ligands, the active sites of molecular docking were determined according to the coordinates of ligands in the target protein complex. Center_x = 42.01, center_y = 7.38, center_z = 38.49, size_x = 43, size_y = 52, size_z = 40, and AutoDock Vina was used for semiflexible docking. The default values were used for the other parameters unless otherwise specified. Finally, Discovery Studio Visualizer 2019 was used to analyze the results of the conformation with the highest score, and PyMOL was used to make correlation diagrams.

### Quantification and Statistical Analysis

Please refer to the figure captions for comprehensive experimental details, encompassing descriptions of the samples (cells, mice), and statistical information. The figure annotations specify the number of mice used, while the cell experiments were conducted with triplicate samples in two independent trials. Data analysis was carried out using GraphPad Prism 8.0 software (GraphPad Software). The data were presented as the mean ± SEM (standard error of the mean). Statistical distinctions were examined using two‐tailed Student's *t*‐tests and one‐way ANOVA (Bonferroni posttest), with significance established at a *p*‐value of ≤ 0.05.

## Conflict of Interest

The authors declare no conflict of interest.

## Author Contributions

Y.W., Q.L., Y.D., C.L., and J.Y. contributed equally to this work. Y. W., G.S., and S.X.H. conceived and designed the experiments. G. W., T. Y., C. L., and Y. W. performed the experiments. T. Y., C. L., Q. L., Y. W., and S.X.H. analyzed the data. T. Y., C. L., Q. L., Y. W., and S.X.H. wrote the manuscript.

## Supporting information

Supporting Information

## Data Availability

The data that support the findings of this study are available from the corresponding author upon reasonable request.

## References

[1] L. Enkler , V. Szentgyörgyi , M. Pennauer , C. Prescianotto‐Baschong , I. Riezman , A. Wiesyk , Nat. Cell Biol. 2023, 25, 1157. 37400497 10.1038/s41556-023-01180-2 PMC10415182

[2] G. Wang , J. Xu , J. Zhao , W. Yin , D. Liu , W. J. Chen , S. X. Hou , Nat. Commun. 2020, 11, 220. 31924786 10.1038/s41467-019-14046-9 PMC6954189

[3] C. Casalou , A. Faustino , D. C. Barral , Small GTPases. 2016, 7, 270. 27589148 10.1080/21541248.2016.1228792 PMC5129889

[4] X. Xie , S. C. Tang , Y. Cai , W. Pi , L. Deng , G. Wu , A. Chavanieu , Y. Teng , OncoTargets Ther. 2016, 7, 58111. 10.18632/oncotarget.11185 PMC5295416 27517156

[5] L. Lang , C. Shay , X. Zhao , Y. Teng , J Exp Clin Cancer Res. 2017, 36, 112. 28830537 10.1186/s13046-017-0583-4 PMC5568197

[6] S. R. Singh , X. Zeng , J. Zhao , Y. Liu , G. Hou , H. Liu , S. X. Hou , Nature. 2016, 538, 109. 27680705 10.1038/nature19788 PMC7798135

[7] P. Aggarwal , Z. Liu , G. Q. Cheng , S. R. Singh , C. Shi , Y. Chen , L. V. Sun , S. X. Hou , Cell Rep. 2022, 39, 110958. 35732115 10.1016/j.celrep.2022.110958 PMC9377423

[8] G. Wang , W. Yin , H. Shin , Q. Tian , W. Lu , S. X. Hou , Nat Aging. 2021, 1, 1024. 37118341 10.1038/s43587-021-00130-7

[9] G. Wang , S. Jin , J. Liu , X.u Li , P. Dai , Y. Wang , S. X. Hou , Natl Sci Rev. 2023, 10, nwad222. 38239560 10.1093/nsr/nwad222 PMC10794899

[10] X. Zeng , S. X. Hou , Development 2015, 142, 644. 25670791 10.1242/dev.113357 PMC4325374

[11] C. Lopez‐Otin , M. A. Blasco , L. Partridge , M. Serrano , G. Kroemer , Cell. 2013, 153, 1194. 23746838 10.1016/j.cell.2013.05.039 PMC3836174

[12] C. Lopez‐Otin , M. A. Blasco , L. Partridge , M. Serrano , G. Kroemer , Cell. 2023, 186, 243. 36599349 10.1016/j.cell.2022.11.001

[13] M. P. Mattson , T. V. Arumugam , Cell Metab. 2018, 27, 1176. 29874566 10.1016/j.cmet.2018.05.011 PMC6039826

[14] H. Ma , W. Fang , Q. Li , Y. Wang , S. X. Hou , Adv. Sci. (Weinh). 2023, 10, e2305089. 37786300 10.1002/advs.202305089 PMC10646219

[15] Y. Ohashi , H. Iijima , N. Yamaotsu , K. Yamazaki , S. Sato , M. Okamura , K. Sugimoto , S. Dan , S. Hirono , T. Yamori , J. Biol. Chem. 2012, 287, 3885. 22158626 10.1074/jbc.M111.316125 PMC3281721

[16] J. M. Zhang , Y. Y. Jiang , Q.‐F.a Huang , X.u‐X. Lu , G. H. Wang , C. L. Shao , M. Liu , Pharmacol Res. 2021, 172, 105800. 34363949 10.1016/j.phrs.2021.105800

[17] C. N. Tseng , C.‐F. Huang , C.‐L. Cho , H.‐W. Chang , C.‐W. Huang , C.‐C. Chiu , Y.‐F. Chang , Molecules. 2013, 18, 10242. 23973996 10.3390/molecules180910242 PMC6270264

[18] N. Prieto‐Dominguez , C. Parnell , Y. Teng , Cells. 2019, 8, 8030255. 10.3390/cells8030255 PMC6468615 30884855

[19] B. E. Housden , M. Muhar , M. Gemberling , C. A. Gersbach , D. Y. R. Stainier , G. Seydoux , S. E. Mohr , J. Zuber , N. Perrimon , Nat. Rev. Genet. 2017, 18, 24. 27795562 10.1038/nrg.2016.118 PMC5206767

[20] T. Achstetter , A. Franzusoff , C. Field , R. Schekman , J. Biol. Chem. 1988, 263, 11711. 3042778

[21] J. Chang , Y. Kim , H. J. Kwon , Nat. Prod. Rep. 2016, 33, 719. 26964663 10.1039/c5np00107b

[22] H. Y. Yoon , J. S. Bonifacino , P. A. Randazzo , Methods Enzymol. 2005, 404, 316. 16413279 10.1016/S0076-6879(05)04028-0

[23] M. Takasugi , Y. Yoshida , E. Hara , N. Ohtani , FEBS J. 2023, 290, 1348. 35106956 10.1111/febs.16381

[24] M. Takasugi , Y. Yoshida , N. Ohtani , Mol. Oncol. 2022, 16, 3333. 35674109 10.1002/1878-0261.13268 PMC9490140

[25] A. M. Mujal , A. J. Combes , A. A. Rao , M. Binnewies , B. Samad , J. Tsui , A. Boissonnas , J. L. Pollack , R. J. Argüello , M. V. Meng , S. P. Porten , M. K. Ruhland , K. C. Barry , V. Chan , M. F. Krummel , Cancer Immunol. Res. 2022, 10, 403. 35181780 10.1158/2326-6066.CIR-21-0588 PMC8982148

[26] Q. Wang , Y. Wang , J. Ding , C. Wang , X. Zhou , W. Gao , H. Huang , F. Shao , Z. Liu , Nature. 2020, 579, 421. 32188939 10.1038/s41586-020-2079-1

[27] P. F. Robbins , M. E. Dudley , J. Wunderlich , M. El‐Gamil , Y. F. Li , J. Zhou , J. Huang , D. J. Powell jr , S. A. Rosenberg , J. Immunol. 2004, 173, 7125. 15585832 10.4049/jimmunol.173.12.7125 PMC2175171

[28] F. Baharom , R. A. Ramirez‐Valdez , K. K. S. Tobin , H. Yamane , C. A. Dutertre , A. Khalilnezhad , G. V. Reynoso , V. L. Coble , G. M. Lynn , M. P. Mulè , A. J. Martins , J. P. Finnigan , X. M. Zhang , J. A. Hamerman , N. Bhardwaj , J. S. Tsang , H. D. Hickman , F. Ginhoux , A. S. Ishizuka , R. A. Seder , Nat. Immunol. 2021, 22, 41. 33139915 10.1038/s41590-020-00810-3 PMC7746638

[29] C. S. Eberhardt , H. T. Kissick , M. R. Patel , M. A. Cardenas , N. Prokhnevska , R. C. Obeng , T. H. Nasti , C. C. Griffith , S. J. Im , X. Wang , D. M. Shin , M. Carrington , Z. G. Chen , J. Sidney , A. Sette , N. F. Saba , A. Wieland , R. Ahmed , Nature. 2021, 597, 279. 34471285 10.1038/s41586-021-03862-z PMC10201342

[30] G. Galletti , G. D. Simone , E. M. C. Mazza , S. Puccio , C. Mezzanotte , T. M. Bi , A. N. Davydov , M. Metsger , E. Scamardella , G. Alvisi , F. D. Paoli , V. Zanon , A. Scarpa , B. Camisa , F. S. Colombo , A. Anselmo , C. Peano , S. Polletti , D. Mavilio , L. Gattinoni , S. K. Boi , B. A. Youngblood , R. E. Jones , D. M. Baird , E. Gostick , S. Llewellyn‐Lacey , K. Ladell , D. A. Price , D. M. Chudakov , E. W. Newell , et al., Nat. Immunol. 2020, 21, 1552. 33046887 10.1038/s41590-020-0791-5 PMC7610790

[31] S. J. Im , M. Hashimoto , M. Y. Gerner , J. Lee , H. T. Kissick , M. C. Burger , Q. Shan , J. Scott Hale , J. Lee , T. H. Nasti , A. H. Sharpe , G. J. Freeman , R. N. Germain , H. I. Nakaya , H. H. Xue , R. Ahmed , Nature. 2016, 537, 417. 27501248 10.1038/nature19330 PMC5297183

[32] C. S. Jansen , N. Prokhnevska , V. A. Master , M. G. Sanda , J. W. Carlisle , M. Asim Bilen , M. Cardenas , S. Wilkinson , R. Lake , A. G. Sowalsky , R. M. Valanparambil , W. H. Hudson , D. McGuire , K. Melnick , A. I. Khan , K. Kim , Y. M. Chang , A. Kim , C. P. Filson , M. Alemozaffar , A. O. Osunkoya , P. Mullane , C. Ellis , R. Akondy , S. J. Im , A. O. Kamphorst , A. Reyes , Y. Liu , H. Kissick , Nature. 2019, 576, 465. 31827286 10.1038/s41586-019-1836-5 PMC7108171

[33] S. Krishna , F. J. Lowery , A. R. Copeland , E. Bahadiroglu , R. Mukherjee , L. Jia , J. T. Anibal , A. Sachs , S. O. Adebola , D. Gurusamy , Z. Yu , V. Hill , J. J. Gartner , Y. F. Li , M. Parkhurst , B. Paria , P. Kvistborg , M. C. Kelly , S. L. Goff , G. Altan‐Bonnet , P. F. Robbins , S. A. Rosenberg , Science. 2020, 370, 1328. 33303615 10.1126/science.abb9847 PMC8883579

[34] S. Kurtulus , A. Madi , G. Escobar , M. Klapholz , J. Nyman , E. Christian , M. Pawlak , D. Dionne , J. Xia , O. Rozenblatt‐Rosen , V. K. Kuchroo , A. Regev , A. C. Anderson , Immunity. 2019, 50, 181. 30635236 10.1016/j.immuni.2018.11.014 PMC6336113

[35] A. M. van der Leun , D. S. Thommen , T. N. Schumacher , Nat. Rev. Cancer. 2020, 20, 218. 32024970 10.1038/s41568-019-0235-4 PMC7115982

[36] S. E. Schad , A. Chow , L. Mangarin , H. Pan , J. Zhang , N. Ceglia , J. X. Caushi , N. Malandro , R. Zappasodi , M. Gigoux , D. Hirschhorn , S. Budhu , M. Amisaki , M. Arniella , D. Redmond , J. Chaft , P. M. Forde , J. F. Gainor , M. D. Hellmann , V. Balachandran , S. Shah , K. N. Smith , D. Pardoll , O. Elemento , J. D. Wolchok , T. Merghoub , J. Exp. Med. 2022, 219, e20212169. 35604411 10.1084/jem.20212169 PMC9130031

[37] B. Zheng , D. Wang , X. Qiu , G. Luo , T. Wu , S. Yang , Z. Li , Y. Zhu , S. Wang , R. Wu , C. Sui , Z. Gu , S. Shen , S. Jeong , X. Wu , J. Gu , H. Wang , L. Chen , Adv. Sci. (Weinh). 2020, 7, 2000224. 32670760 10.1002/advs.202000224 PMC7341083

[38] M. D. Young , S. Behjati , Gigascience. 2020, 9, 151. 10.1093/gigascience/giaa151 PMC7763177 33367645

[39] G. X. Y. Zheng , J. M. Terry , P. Belgrader , P. Ryvkin , Z. W. Bent , R. Wilson , S. B. Ziraldo , T. D. Wheeler , G. P. McDermott , J. Zhu , M. T. Gregory , J. Shuga , L. Montesclaros , J. G. Underwood , D. A. Masquelier , S. Y. Nishimura , M. Schnall‐Levin , P. W. Wyatt , C. M. Hindson , R. Bharadwaj , A. Wong , K. D. Ness , L. W. Beppu , H. J. Deeg , C. McFarland , K. R. Loeb , W. J. Valente , N. G. Ericson , E. A. Stevens , J. P. Radich , et al., Nat. Commun. 2017, 8, 14049. 28091601 10.1038/ncomms14049 PMC5241818

[40] S. L. Wolock , R. Lopez , A. M. Klein , Cell Syst. 2019, 8, 281. 30954476 10.1016/j.cels.2018.11.005 PMC6625319

[41] Y. Hao , S. Hao , E. Andersen‐Nissen , W. M. Mauck , S. Zheng , A. Butler , M. J. Lee , A. J. Wilk , C. Darby , M. Zager , P. Hoffman , M. Stoeckius , E. Papalexi , E. P. Mimitou , J. Jain , A. Srivastava , T. Stuart , L. M. Fleming , B. Yeung , A. J. Rogers , J. M. McElrath , C. A. Blish , R. Gottardo , P. Smibert , R. Satija , Cell. 2021, 184, 3573. 34062119 10.1016/j.cell.2021.04.048 PMC8238499

[42] I. Korsunsky , N. Millard , J. Fan , K. Slowikowski , F. Zhang , K. Wei , Y. Baglaenko , M. Brenner , P.‐R. Loh , S. Raychaudhuri , Nat. Methods. 2019, 16, 1289. 31740819 10.1038/s41592-019-0619-0 PMC6884693

[43] X. Qiu , Q. Mao , Y. Tang , L. Wang , R. Chawla , H. A Pliner , C. Trapnell , Nat. Methods. 2017, 14, 979. 28825705 10.1038/nmeth.4402 PMC5764547

[44] M. Li , X. Zhang , K. S. Ang , J. Ling , R. Sethi , N. Y. S. Lee , F. Ginhoux , J. Chen , Nucleic Acids Res. 2022, 50, D596. 34791375 10.1093/nar/gkab1020 PMC8728243

[45] C. S. McGinnis , L. M. Murrow , Z. J. Gartner , Cell Syst. 2019, 8, 329. 30954475 10.1016/j.cels.2019.03.003 PMC6853612

