# Supplementary material for: Novel Arf1 Inhibitors Drive Cancer Stem Cell Aging and Potentiate Anti‐Tumor Immunity
Source: Adv Sci (Weinh). 2024 Sep 3;11(39):2404442. doi: 10.1002/advs.202404442 (PMC11497069; doi:10.1002/advs.202404442)
Supplement: Supplementary file 1 — Supporting Information [file ADVS-11-2404442-s001.pdf]

ADVANCED SCIENCE
Open Access

Supporting Information

for *Adv. Sci.*, DOI 10.1002/advs.202404442

Novel Arf1 Inhibitors Drive Cancer Stem Cell Aging and Potentiate Anti-Tumor Immunity

Yuetong Wang, Qiaoming Li, Yahui Ding, Chenfei Luo, Jun Yang, Na Wang, Ning Jiang, Tiange Yao, Guohao Wang, Guoming Shi and Steven X. Hou**

Supplementary Materials for

Novel Arf1 inhibitors drive cancer stem cell aging and activate a trans-cellular anti-tumor immunity

Yuetong Wang[1, #], Qiaoming Li[1, #], Yahui Ding[1, #], Chenfei Luo[1, #], Jun Yang[1, #], Na Wang[1], Ning Jiang[1], Tiange Yao[1], Guohao Wang[2], Guoming Shi[1, *], Steven X. Hou[1, 3, *]

[1]Department of Cell and Developmental Biology at School of Life Sciences, State Key Laboratory of Genetic Engineering, Institute of Metabolism and Integrative Biology, Human Phenome Institute, Department of Liver Surgery and Transplantation of Liver Cancer Institute at Zhongshan Hospital, Fudan University, Shanghai 200438, China

[2]The Basic Research Laboratory, Center for Cancer Research, National Cancer Institute at Frederick, National Institutes of Health, Frederick, MD 21702, USA

[#]The first five authors made equal contribution

[3]Leading Contact

*Correspondence: shigm@usx.edu.cn， stevenhou@fudan.edu.cn (S.X.H.)

Concise running title: New Arf1 inhibitors promote anti-tumor immunity

This PDF file includes:

Figures S1 to S10

Table S1

Table S2

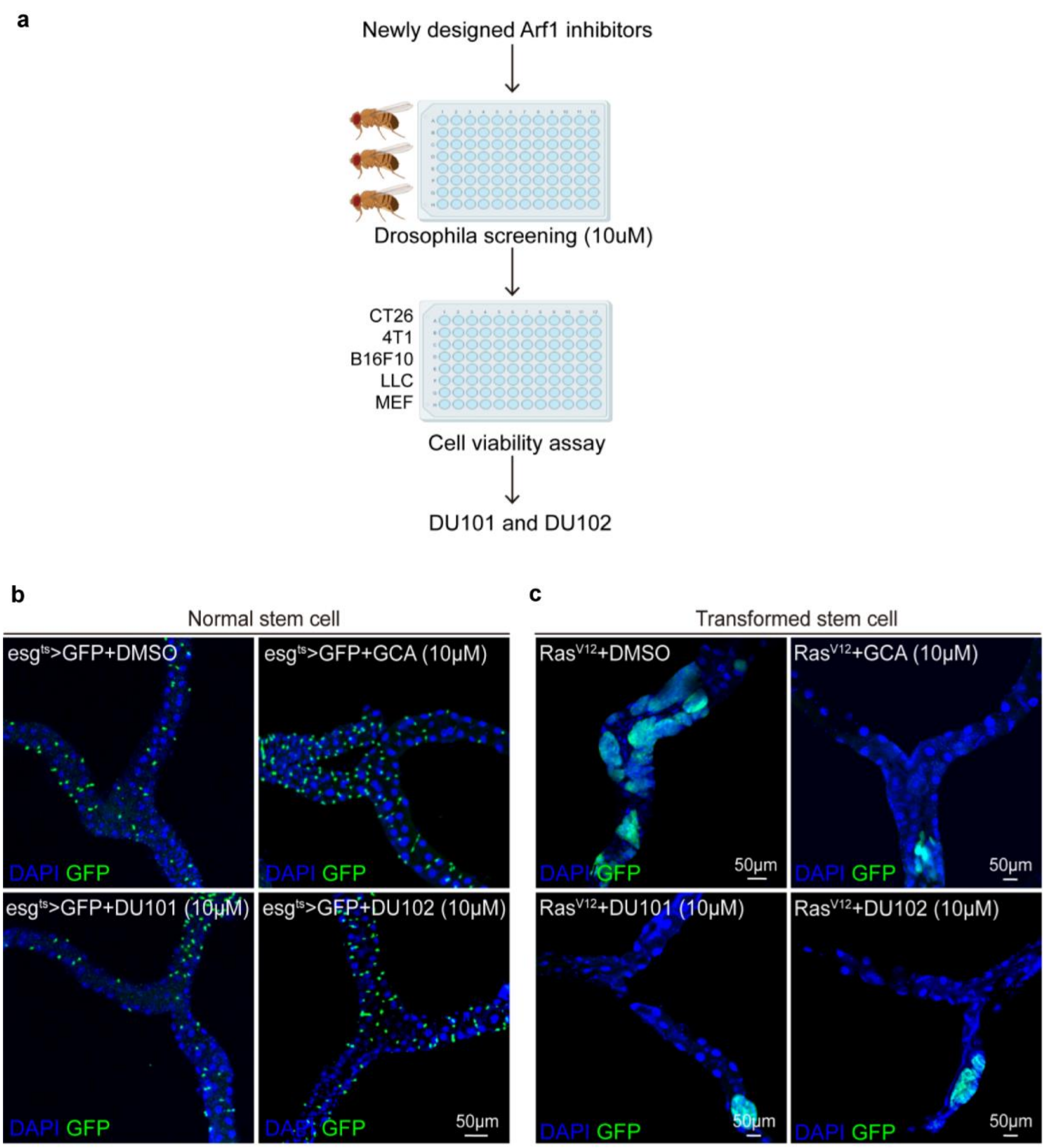

Figure S1. The strategy for screening new Arf1 inhibitors in Drosophila.

(a) The scheme of new Arf1 inhibitors screening strategy in Drosophila and mouse cancer cells.

(b) The GCA and new compounds DU101 and DU102 did not kill normal stem cells in Drosophila. Flies with normal stem cells or with Ras^{V12}-transformed RNSC tumors were given normal food + 10 μM GCA or normal food + 10 μM DU101or normal food + 10 μM DU102. GFP marks stem cell tumors. Scale bars = 50 μm.

(c) The GCA and new compounds DU101 and DU102 selectively killed the Ras^{V12}- transformed stem cells. Flies with normal stem cells or with Ras^{V12}-transformed RNSC tumors were given normal food + 10 μM GCA or normal food + 10 μM DU101 or normal food + 10 μM DU102.

GFP marks stem cell tumors. Scale bars = 50 μm.

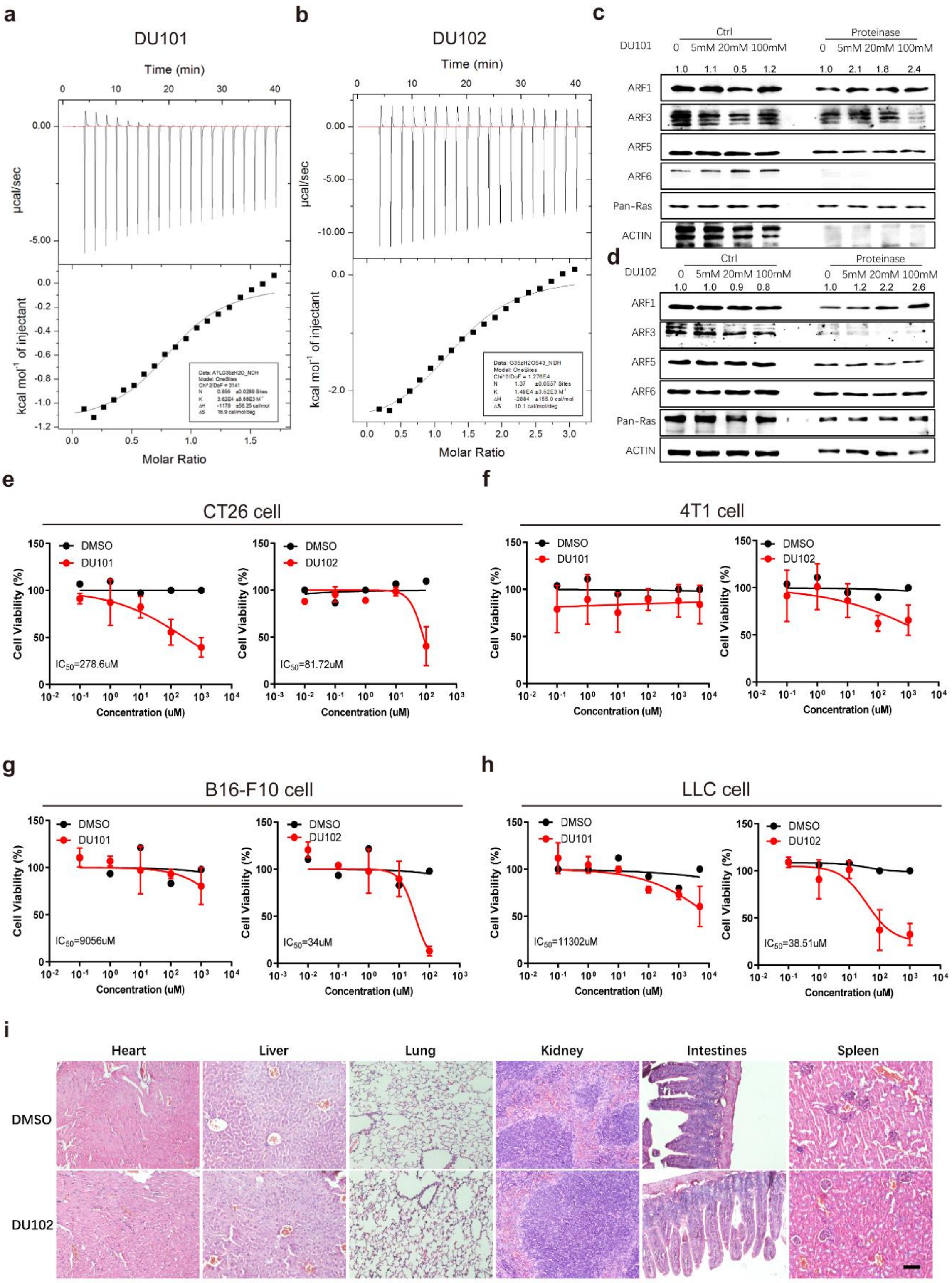

Figure S2. DU101 and DU102 are specific inhibitors of Arf1 and have low toxicity.

(a-b) Microcalorimetry data. Upper panel: ITC thermograms showing titration of the Sec7

domain (0.362 μM) by DU101 (3 μM) (a) or DU102 (3.62 μM) (b) at 25 °C. Lower panel: the plots showing the heat released from the above titration for each ligand. The dotted lines represent the best least square fits for the experimental data using a one site model.

(c-d) Western blot showed the indicated protein levels in total cell lysis. CT26 cell total lysis were collected and incubated with DU101 or DU102 for 30min at room temperature protected from light. Then proteinases were added and the lysis were digested for 30min at room temperature. After digestion, the cell lysis was used for western blot. Experiments were repeated 3 times.

(e-h) The cell viabilities of treatments of DMSO and DU101 or DU102 on mouse CT26 colon cancer cell (e), mouse 4T1 breast cancer cell (f), mouse B16-F10 melanoma cell (g) and mouse LLC cell (h). Data are shown as mean ± SEM.

(i) Mice were treated with DMSO or 5mg/kg DU102 for 14 days (n=5). Then different organs were collected for hematoxylin-eosin staining. Representative photographs were showed. Scale bar indicated 50μm.

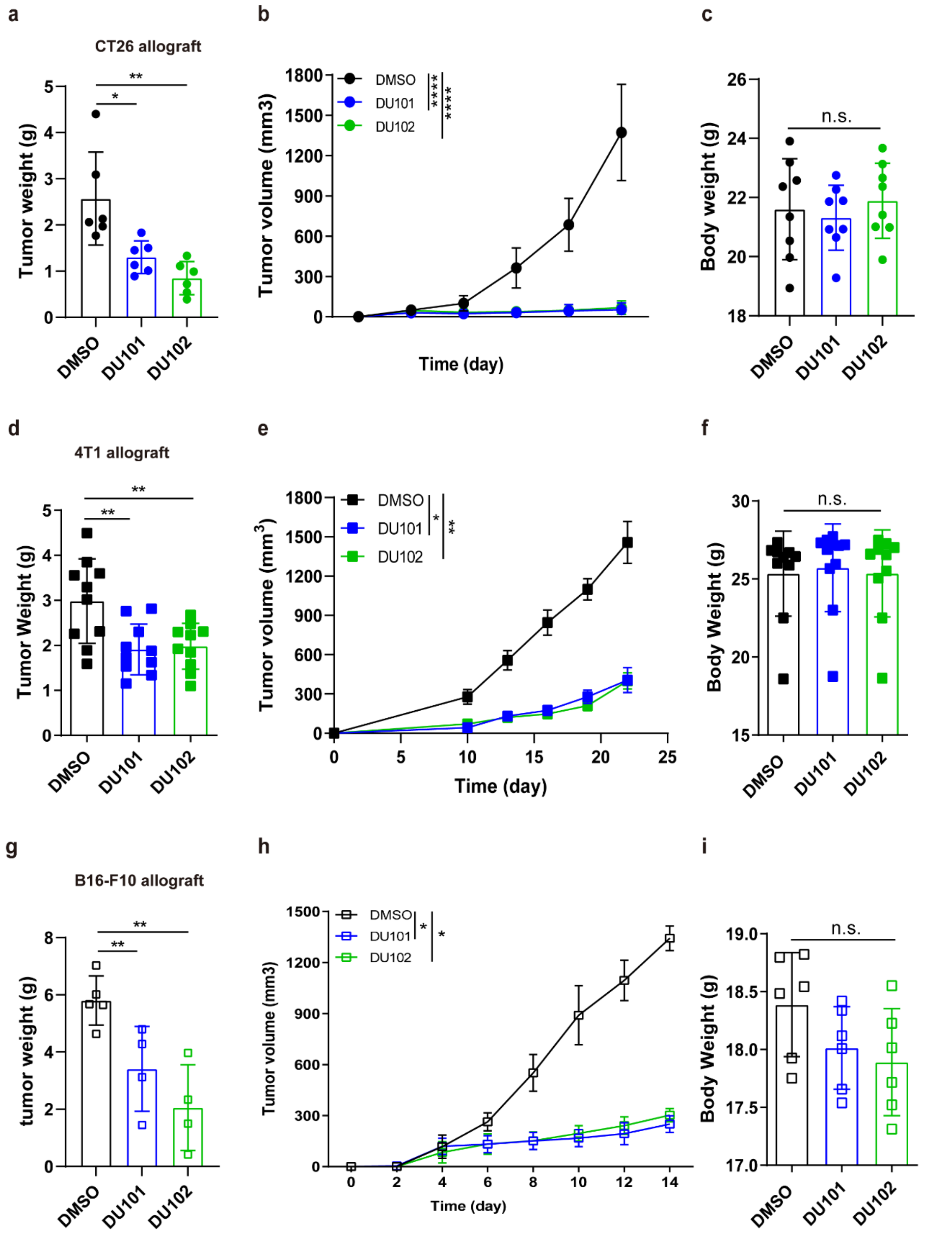

Figure S3. The new Arf1 inhibitors induced tumor regression in mouse tumor models of colon, breast and melanoma.

(a-c) Tumor weights, tumor volumes, and body weights of BALB/c mice transplanted with murine colon tumor CT-26 cells and treated with the indicated reagents. (n=6 mice each group).

(d-f) Tumor weights, tumor volumes, and body weights of BALB/c mice transplanted with murine breast tumor 4T1 cells and treated with the indicated reagents. (n=10 mice each group).

(g-i) Tumor weights, tumor volumes, and body weights of C57BL/6J mice transplanted with murine melanoma B16-F10 cells and treated with the indicated reagents. (n=6 mice each group).

Data are shown as mean ± SEM. Student's t test. *P< 0.05, **P< 0.01, ****P< 0.0001; n.s., not significant.

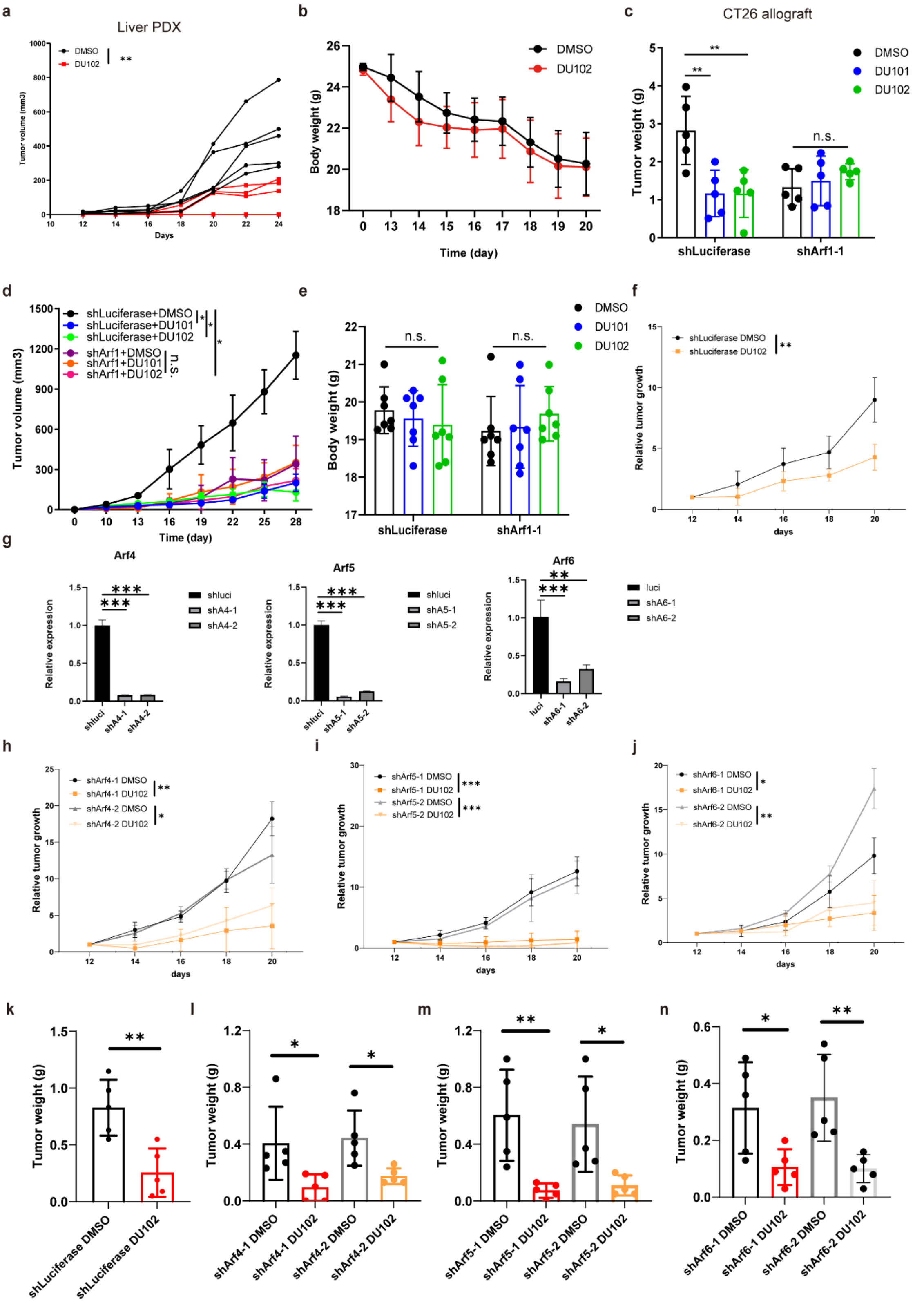

Figure S4. The new Arf1 inhibitors induced tumor regression in the liver tumor PDX model and specifically target Arf1.

(a) Tumor volumes of the humanized immune and liver tumor PDX mice treated with the indicated reagents. (n=5 mice each group).

(b) Body weights of the humanized immune and liver tumor PDX mice treated with the indicated reagents. (n=3 mice each group).

(c-e) Tumor weights (c), tumor volumes (d), and body weights (e) of BALB/c mice transplanted with murine colon tumor CT-26 cells that were transfected with shArf1 or shLuciferase and treated with the indicated reagents. (n=7 mice each group).

(g) Relative expression of indicated genes in CT-26 cells that were transfected with shArf4, shArf5, shArf6 or shLuciferase detected by Q-PCR. (n=3).

(h-j) Relative tumor volumes of BALB/c mice transplanted with murine colon tumor CT-26 cells that were transfected with shArf4, shArf5, shArf6 or shLuciferase and treated with the indicated reagents. (n=5 mice each group).

(k-n) Tumor weight of BALB/c mice transplanted with murine colon tumor CT-26 cells that were transfected with shArf4, shArf5, shArf6 or shLuciferase and treated with the indicated reagents. (n=5 mice each group).

Data are shown as mean ± SEM. Student's t test. *P< 0.05, **P< 0.01; n.s., not significant.

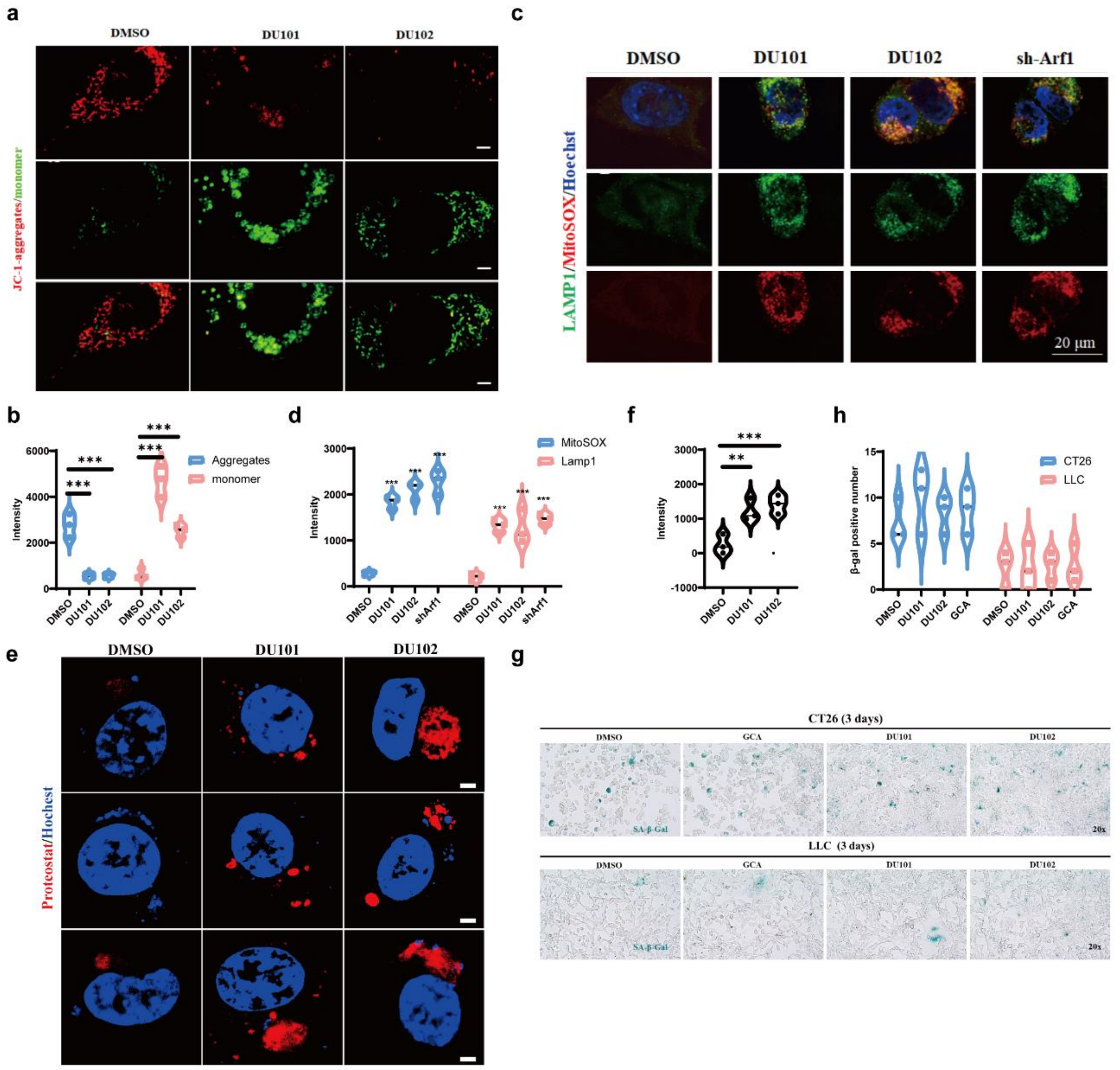

Figure S5. The new Arf1 inhibitors induced tumor cell aging cascade.

(a) Treatment of CT26 cells with DU101 and DU102 dramatically reduced the mitochondrial membrane potential. When the mitochondrial membrane potential is high, JC-1 aggregates in the matrix of mitochondria to form polymers (J-aggregates), which can produce red fluorescence. When the mitochondrial membrane potential is low, JC-1 cannot accumulate in the mitochondrial matrix, and JC-1 is a monomer and can produce green fluorescence. Scale Bars: 5 μM.

(b) Quantification for fluorescence intensity of (a).

(c) Treatment of CT26 cells with DU101 and DU102 or Arf1 gene knockdown (sh-Arf1) dramatically increased ROS production as assayed by elevated Mitosox level and induced expression of the lysosome protein LAMP1. Scale Bar: 20 μM.

(d) Quantification for fluorescence intensity of (c).

(e) Treatment of CT26 cells with Arf1 inhibitors DU101 and DU102 dramatically increased protein aggregation as illustrated by increased staining of Proteostat dye. Proteostat dye becomes highly fluorescent upon binding to the amyloid-type b-sheet tertiary structure of protein aggregates. Scale Bars: 5 μM.

(f) Quantification for fluorescence intensity of (e).

(g) Treatment of CT26 cells with Arf1 inhibitors GCA or DU101 or DU102 did not change expression of Senescence-associated β-galactosidase (SA-β-gal), suggesting that the Arf1-ablation-induced aging is different from the commonly known cellular senescence.

(h) Quantification for positive staining of β-galactosidase (SA-β-gal) (g).

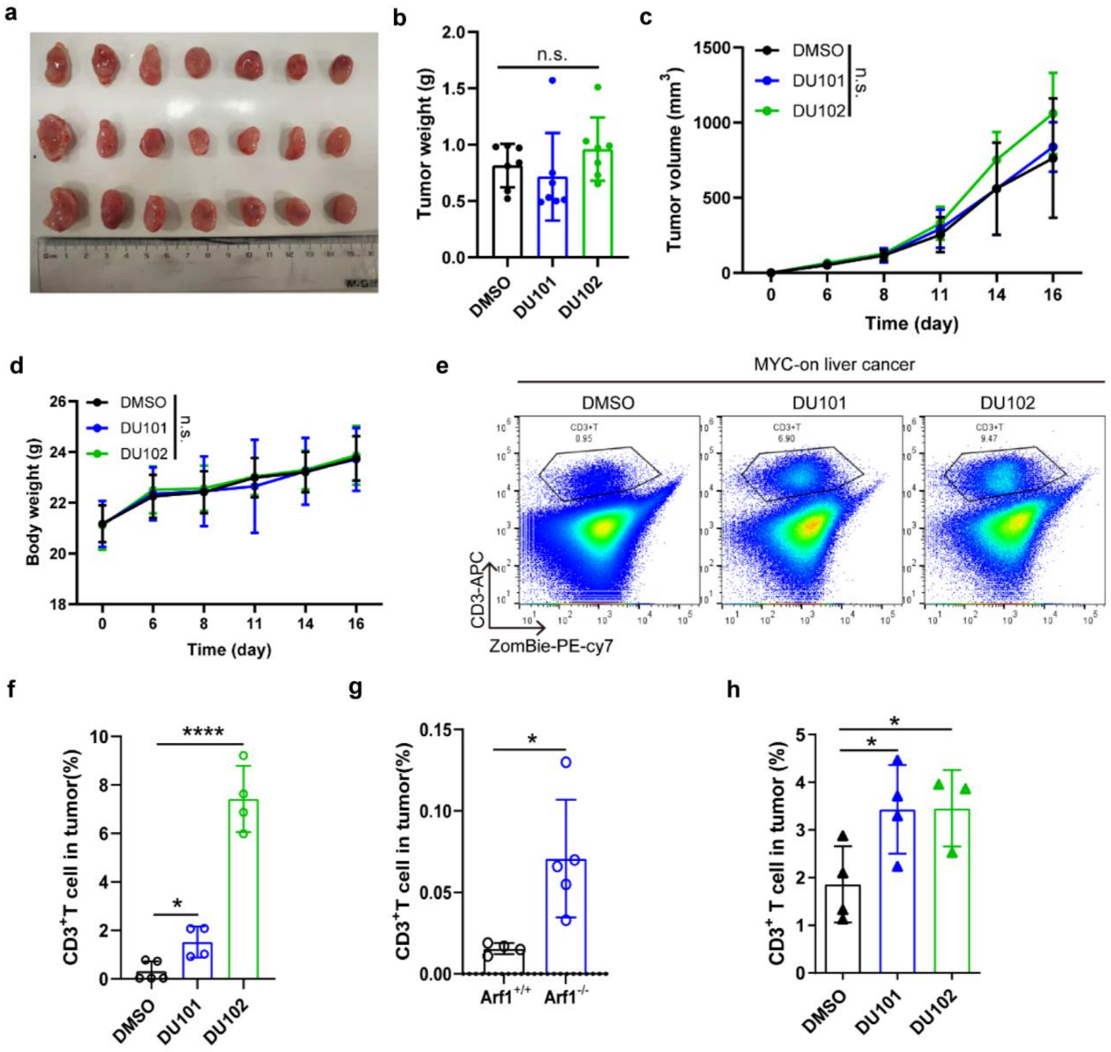

Figure S6. DU101 and DU102 induced tumor regression by increasing tumor infiltrated T cells.

(a-b) Representative tumor images (a) and tumor weights (b) of tumors from nude mice that were transplanted with CT26 cells and treated with the indicated reagents. (n=7 mice each group).

(c) The curves showed tumor volume of tumors from nude mice that were transplanted with CT26 cells and treated with the indicated reagents. (n=7 mice each group). Data are shown as mean ± SEM. n.s., no significance.

(d) The curves showed body weights of nude mice that were transplanted with CT26 cells and treated with the indicated reagents. (n=7 mice each group). Data are shown as mean ± SEM.

n.s.= no significance.

(e) FACS plots of $CD3^+$ T cells in MYC-ON liver cancer treated with DMSO or DU101 or DU102. (n=5 mice each group)

(f) Quantification for $CD3^+$ T cells in (e).

(g) FACS analysis of $CD3^+$ T cells in Arf1-knockout MYC-on liver tumor. (n=5 mice each group)

(h) The proportion of $CD3^+$ T cells in hepa1-6 allograft treated with DMSO or DU101 or DU102. (n=4 mice each group).

Data are shown as mean ± SD. Student’s t test. *P< 0.05, ****P< 0.0001.

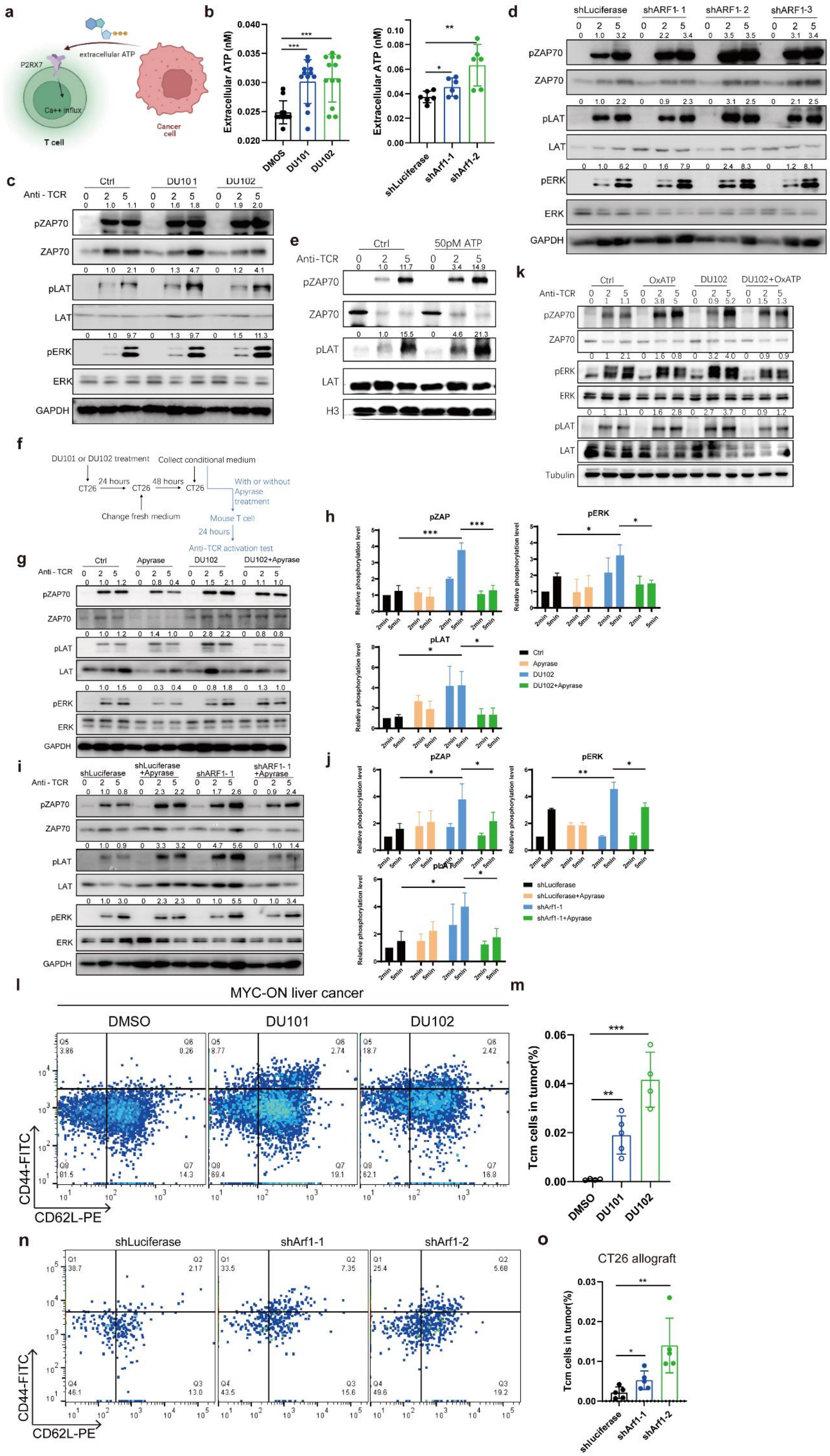
a
extracellular ATP
P2RX7
Ca++ influx
T cell
Cancer cell
b
Extracellular ATP (nM)
DMOS
DU101
DU102
shLuciferase
shArf1-1
shArf1-2
c
Ctrl
DU101
DU102
Anti - TCR
pZAP70
ZAP70
pLAT
LAT
pERK
ERK
GAPDH
d
shLuciferase
shARF1-1
shARF1-2
shARF1-3
e
50pM ATP
H3
f
DU101 or DU102 treatment
Collect conditional medium
24 hours
48 hours
CT26
Change fresh medium
With or without Apyrase treatment
Mouse T cell
24 hours
Anti-TCR activation test
g
Apyrase
DU102+Apyrase
h
pZAP
pERK
pLAT
Relative phosphorylation level
Ctrl
Apyrase
DU102
DU102+Apyrase
i
shLuciferase+Apyrase
shARF1-1
shARF1-1+Apyrase
j
shLuciferase
shLuciferase+Apyrase
shArf1-1
shArf1-1+Apyrase
k
OxATP
DU102+OxATP
Tubulin
l
MYC-ON liver cancer
DMSO
DU101
DU102
CD44-FITC
CD62L-PE
m
Tcm cells in tumor(%)
DMSO
DU101
DU102
n
shLuciferase
shArf1-1
shArf1-2
CD44-FITC
CD62L-PE
o
CT26 allograft
Tcm cells in tumor(%)
shLuciferase
shArf1-1
shArf1-2

Figure S7. Arf1 knockdowns induced T cell activation though extracellular ATP.

(a) A proposed model of the interaction between extracellular ATP from tumor cells and P2RX7 receptor on T cells.

(b) The levels of extracellular ATP from CT26 cells were measured after treatments with the indicated reagents for 24 hours. And the levels of extracellular ATP were measured from CT26 cells that were transfected with shLuciferase or shArf1. (n=6)

(c) T cell activities were measured by examining the phosphorylation level of ZAP70 (pZAP70), LAT (pLAT) and ERK (pERK) after culturing with the conditional medium from CT-26 cells treated with the indicated reagents for 24 hours. Experiment was repeated 3 times.

(d) T cell activities were measured by examining phosphorylation of ZAP70 (pZAP70), LAT (pLAT) and ERK (pERK) after culturing with cell medium collected from control (shLuciferase) or Arf1-deficient (shArf1) CT26 cells.

(e) T cell activities were measured by examining phosphorylation of ZAP70 (pZAP70), LAT (pLAT) and ERK (pERK) after adding 50pM ATP into culture medium.

(f) Scheme showing the procedure of conditional medium co-culture experiment.

(g) The T cell activation induced by conditional medium from CT-26 cells treated with the new Arf1 inhibitors was abolished after adding Apyrase (an ATP hydrolytic enzyme) to the experiments in (f). Experiment was repeated 3 times.

(h) Bar plot showing the relative gray scale level changes of ZAP70 (pZAP70), LAT (pLAT) and ERK (pERK) of (g). n=3

(i) The T cell activation induced by Arf1 knockdown was abolished after adding Apyrase (an ATP hydrolytic enzyme).

(j) Bar plot showing the relative gray scale level changes of ZAP70 (pZAP70), LAT (pLAT) and ERK (pERK) of (i). n=3

(k) T cell activities were measured by examining phosphorylation of ZAP70 (pZAP70), LAT (pLAT) and ERK (pERK) in T cells treated with or without OxATP.

(l) Representative flow cytometry plots showing frequencies of the $CD44^{+}$ $CD62L^{+}$ central

memory (T_{CM}) cells in liver tumors of MYC-ON mice after treatments with the indicated reagents. (n = 5 per genotype).

(m) Quantification of the data in (k).

(n) Representative flow cytometry plots showing frequencies of the $CD44^+$ $CD62L^+$ central memory (Tcm) cells in mouse allografts transplanted with murine colon tumor CT-26 cells that were transfected with shArf1 or shLuciferase. (n=8 mice each group).

(o) Bar plot showing the percentage of the $CD44^+$ $CD62L^+$ central memory (Tcm) cells in mouse allografts transplanted with murine colon tumor CT-26 cells that were transfected with shArf1 or shLuciferase. (n=8 mice each group).

Data were shown as mean ± SEM. Student's t test. *P< 0.05, **P< 0.01, ***P< 0.001; n.s., not significant.

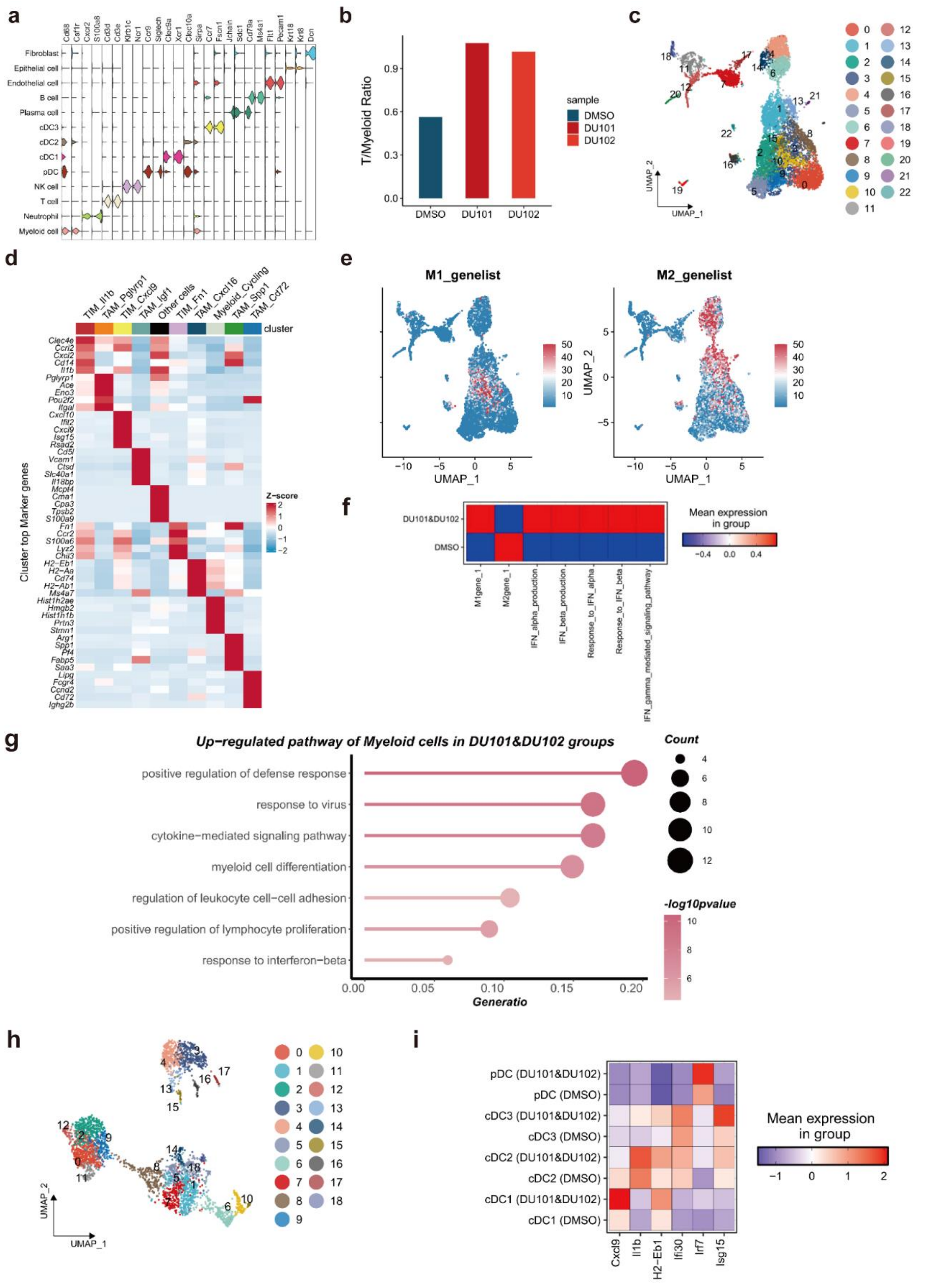

Figure S8. Single cell RNA sequencing revealed reprogrammed myeloid cells after treatment with the new Arf1 inhibitors.

(a) Stacked violin plots showing markers used for annotation of immune cells.

(b) Ratio of T cells versus myeloid cells in each treatment group.

(c) Uniform manifold approximation and projection (UMAP) of Myeloid cells in TME.

(d) Heatmap showing top 5 feature genes of each subtype of Myeloid cells.

(e) Feature plot showing the expression of macrophage M1 polarization and macrophage M2 polarization gene sets in Myeloid cells.

(f) Heatmap showing expression of macrophage M1 polarization and macrophage M2 polarization gene sets in Myeloid cells in each treatment group.

(g) GO analysis of the up-regulated genes of Myeloid cells in DU101 and DU102 treatment groups.

(h) Uniform manifold approximation and projection (UMAP) of DCs in TME.

(i) Heatmap showing expression of selected genes in different DC subtypes in each treatment group.

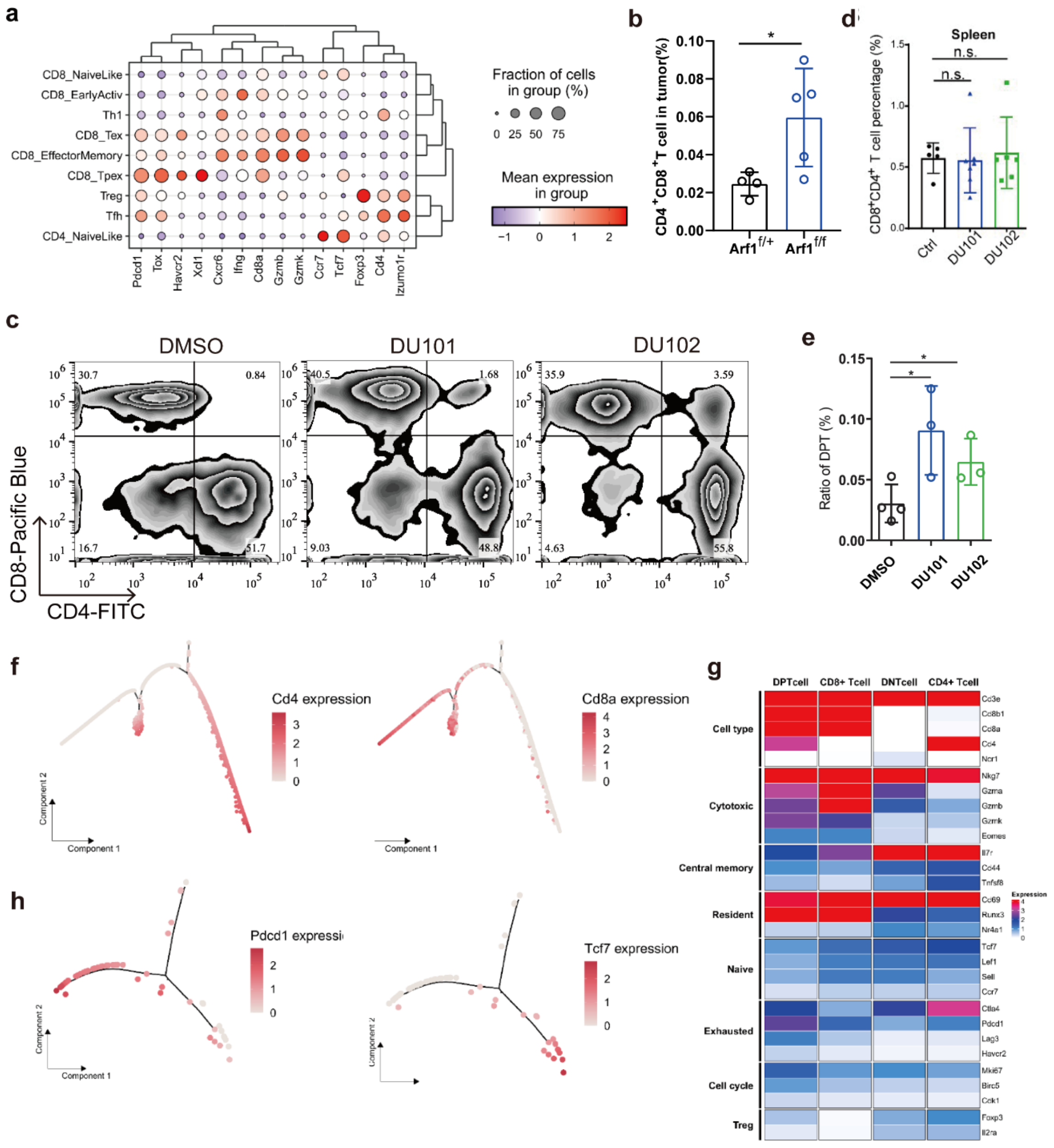

Figure S9. The new Arf1 inhibitors reprogrammed T cells with superior anti-tumor activity.

(A) Dot plots showing expression of markers used for T cell subtype annotation.

(B) Quantification of the percentage of CD8^{+} CD4^{+} T cells in MYC-ON mice after Arf1 knockdown (Arf1$^{-/-}$) in comparison with those in tumors of control mice (Arf1$^{+/+}$). (n=5 mice).

(C) Flow cytometry analysis of CD8^{+} CD4^{+} T cells in liver tumors of MYC-ON mice after

DMSO, DU101 or DU102 treatment. (n=5 mice).

(D) Quantification of the percentage of $CD8^+$ $CD4^+$ T cells in spleens of MYC-ON mice after DMSO, DU101 or DU102 treatment. (n=5 mice).

(E) Quantification of the percentage of $CD8^+$ $CD4^+$ T cells in liver tumors of MYC-ON mice after DMSO, DU101 or DU102 treatment. (n=5 mice).

(F) Plotting the expression of Cd4 and Cd8a on the T cell trajectory.

(G) Heatmap showing the expression pattern of marker genes in indicated T cell subpopulation.

(H) Plotting the expression of Tcf7 and Pdcd1 on the DPT cell trajectory.

Data are shown as mean ± SEM. Student's t test. *$P < 0.05$, n.s., not significant.

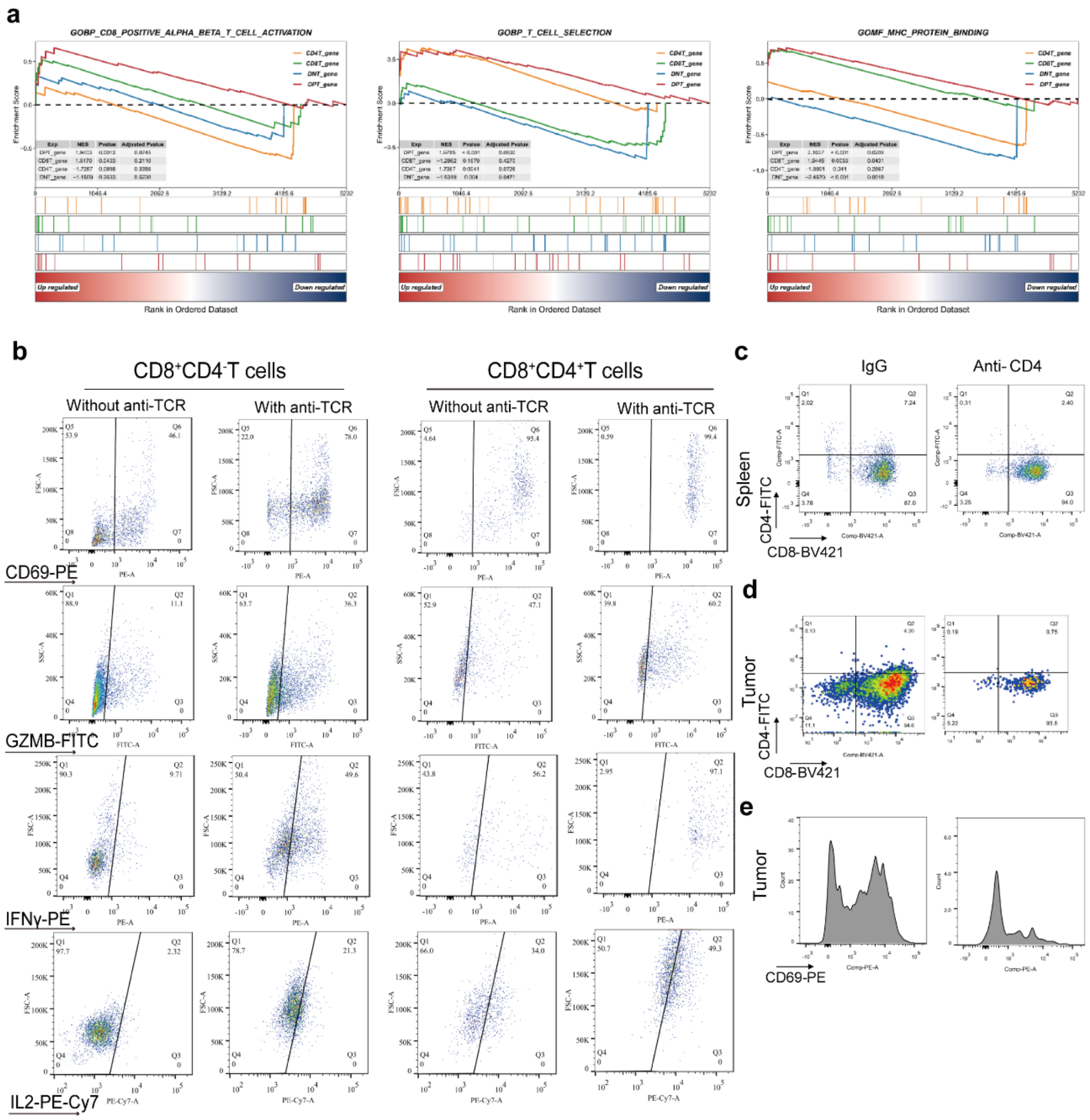

Figure S10. CD8$^+$ CD4$^+$ T cells exhibited strong cytotoxic function in vivo.

(a) GSEA analysis of the top feature genes of CD8 SPT, CD4 SPT, DPT, and DNT cells.

(b) Flow cytometric analysis of CD69, GZMB, IFNγ and IL-2 in CD8$^+$ CD4$^-$ or CD8$^+$ CD4$^+$ T cells after stimulation with anti-CD3 and anti-CD28 antibodies for 24 hours. (n=3).

(c) Flow cytometric analysis of CD8$^+$ CD4$^+$ T cells isolated from the spleen. (n=5).

(d) Flow cytometric analysis of CD8$^+$ CD4$^+$ T cells isolated from tumors. (n=5).

(e) Flow cytometric analysis of CD69$^+$ T cells isolated from tumors. (n=5).

Table S1 Primer list

Primer	Sequence
shSphK1-1-F-Mus	CCGGGCACCCAAACTACCTTTGGATCTCGAGATCCAAAGGT AGTTTGGGTGCTTT TTG
shSphK1-1-R-Mus	AATTCAAAAAGCACCCAAACTACCTTTGGATCTCGAGATCC AAAGGTAGTTTGGG TGC
shSphK1-2-F-Mus	CCGGCTACTCTGTCTAGGCTGAGATCTCGAGATCTCAGCCTA GACAGAGTAGTTT TTG
shSphK1-2-R-Mus	AATTCAAAAACTACTCTGTCTAGGCTGAGATCTCGAGATCTC AGCCTAGACAGAG TAG
Q-mSPHK1-F	ACTGATACTCACCGAACGGAA
Q-mSPHK1-R	CCATCACCGGACATGACTGC
Q-mACTIN-F	GGCTGTATTCCCCTCCATCG
Q-mACTIN-R	CCAGTTGGTAACAATGCCATGT
Q-mGAPDH-F	AGGTCGGTGTGAACGGATTTG
Q-mGAPDH-R	GGGGTCGTTGATGGCAACA
Q-18S-F	GTAACCCGTTGAACCCCATT
Q-18S-R	CCATCCAATCGGTAGTAGCG
Q-mCD3-F	CTGCTACACACCAGCCTCAA
Q-mCD3-R	CATCAGCAAGCCCAGAGTGA
Q-mCD4-F	CCAGACAGTGTTCCTGGCTT

Q-mCD4-R	TGCCTGGCGCTGTTGG
Q-mCD8-F	CAAATGTCCCAGGCCGCTA
Q-mCD8-R	TCCTGGCGGTGCCATTTTAC
P23-GGA3-F	CCATTTCAGGTGTCGTGAAGCGGCCGCCCACCATGAAGAAC T
P23-GGA3-R	TTGCTCTAGACTCGAGCTAGCCTCGAGTAGGTTCCCCCACTG
M-shARF1-F-1	CCGG GGAATATCTTTGCAAACCTCT CTCGAG AGAGGTTTGCAAAGATATTCC TTTTTG
M-shARF1-R-1	AATTCAAAAA GGAATATCTTTGCAAACCTCT CTCGAG AGAGGTTTGCAAAGATATTCC
M-shARF1-F-2	CCGG GCGAAATTGTGACCACCATTC CTCGAG GAATGGTGGTCACAATTTCGC TTTTTG
M-shARF1-R-2	AATTCAAAAA GCGAAATTGTGACCACCATTC CTCGAG GAATGGTGGTCACAATTTCGC

Table S2 Key resource table

REAGENTS or RESOUR	SOURCE	IDENTIFIER
**Antibodies**		
Rat anti-mouse CD3-APC	Biolegend	Cat# 100236; RRID: AB_2561456;
Rat anti-mouse CD4-FITC	Biolegend	Cat# 100405; RRID: AB_312690;
Rat anti-mouse CD8-Pacific Blue	Biolegend	Cat# 100725; RRID: N/A;
Armenian Hamster anti-mouse CCR5-PerCP/Cyanine5.5	Biolegend	Cat# 107015; RRID: AB_2616985;
Zombie NIR™ Fixable Viability Kit	Biolegend	Cat# 423106; RRID: N/A;
Rat anti-mouse CD45-PE	Biolegend	Cat# 103105; RRID: AB_312970;
Armenian Hamster anti-mouse CD69-PE	Biolegend	Cat# 104507; RRID: AB_313110;
Mouse anti-mouse/human Granzyme B-FITC	Biolegend	Cat# 372205; RRID: AB_2687029;
Rat anti-mouse IL-2- PE/Cyanine7	Biolegend	Cat# 503831; RRID: AB_2561749;
Rat anti-mouse IFNγ-PE	Biolegend	Cat# 505807; RRID: N/A;
Rabbit anti-mouse TCF1-PE	Cell Signaling Technology	Cat# 14456S; RRID: AB_2798483;
Rat anti-mouse CD62L-PE	Biolegend	Cat# 104407; RRID: AB_313094;
Rat anti-mouse/human CD44-FITC	Biolegend	Cat# 103022; RRID: AB_493685;
Rat anti-mouse CD326 (Ep-CAM)-FITC	Biolegend	Cat#118207; RRID: AB_1134106;
Rat anti-mouse CD279 (PD-1)-APC	Biolegend	Cat# 135210; RRID: AB_2159183;
Mouse anti-human CD3-APC	Biolegend	Cat# 317318; RRID: AB_1937212;
Mouse anti-human CD4-Brilliant Violet 605™	Biolegend	Cat# 317438; RRID: N/A;
Mouse anti-human CD8-Percp/cy5.5	Biolegend	Cat# 344709; RRID: AB_2044009;
Mouse anti-human TCF1-Alexa Fluor® 647	Biolegend	Cat# 655203; RRID: N/A;
Mouse anti-human PD-1-FITC	Biolegend	Cat# 329903; RRID: AB_940477;

Mouse anti-human CD45RO-Brilliant Violet 421™	Biolegend	Cat# 304223; RRID: N/A;
Mouse anti-human CD62L-FITC	Biolegend	Cat# 304803; RRID: AB_314463;
Rabbit anti-CD4 antibody	Abcam	Cat# ab288724; RRID: N/A;
Mouse anti-CD8 antibody	Thermo Fisher Scientific	Cat# MA1-7632; RRID: AB_1073402;
Donkey anti-rabbit IgG H&L -Alexa Fluor® 488	Abcam	Cat# ab150061; RRID: AB_2571722;
Donkey anti-mouse IgG H&L -Alexa Fluor® 568	Abcam	Cat# ab175700; RRID: N/A;
Goat anti-rabbit IgG H&L -Alexa Fluor® 568	Abcam	Cat# ab175471; RRID: AB_2576207;
Goat anti-mouse IgG H&L -Alexa Fluor® 488	Abcam	Cat# ab150113; RRID: AB_2576208;
Goat anti-mouse IgG H&L-HRP	Abcam	Cat# ab6789; RRID: AB_955439;
Goat anti-rabbit IgG H&L-HRP	Abcam	Cat# ab6721; RRID: AB_955447;
Rabbit anti-Arf1 antibody	Thermo Fisher Scientific	Cat# PA1-127; RRID: AB_2539892;
Rabbit anti-H3 antibody	Beyotime	Cat# AF7101; RRID: N/A;
Mouse anti-GAPDH antibody	Beyotime	Cat# AF2819; RRID: N/A;
Mouse anti-IgG antibody	Cell Signaling Technology	Cat# 5415S; RRID: AB_10829607;
anti-mouse CD3ε antibody	InVivoMAb	Cat# BE0001-1; RRID: AB_1107634;
anti-mouse CD28 antibody	InVivoMAb	Cat# BE0015-1; RRID: AB_1107624;
anti-phosphorLAT antibody	Cell Signaling Technology	Cat# 20170; RRID: N/A;
anti-LAT antibody	Cell Signaling Technology	Cat# 45533; RRID: N/A;
anti-phosphorERK antibody	Cell Signaling Technology	Cat# 9109; RRID: AB_2297442;
anti-ERK antibody	Cell Signaling Technology	Cat# 9102; RRID: AB_330744;
anti-phosphorZAP70 antibody	Cell Signaling Technology	Cat# 2701; RRID: AB_331600;
anti-ZAP70 antibody	Cell Signaling Technology	Cat# 2705; RRID: AB_2273231;
Rat IgG2b isotype control	InVivoMAb	Cat# BP0090; RRID: N/A;
anti-mouse CD4 antibody	InVivoMAb	Cat# BP0003; RRID: N/A;
**Bacterial and virus strains**		

Trans5α	Transgen	Cat# CD201-01
TransIT®-2020	Mirusbio	Cat# MIR 5400
**Biological samples**		
Patient liver cancer tissue	Zhongshan Hospital-Fudan University	N/A
**Primers**		
Primers	Table S1	N/A
**Chemicals, peptides, and recombinant proteins**		
Brefeldin A	Selleck	Cat# S7046
DAPI	Sigma-Aldrich	Cat# D9542
Puromycin Dihydrochloride	Beyotime	Cat# ST551
Recombinant Human IL-2 protein	Genescript	Cat# Z00368
D-Luciferin potassium salt	Beyotime	Cat# ST196
Tamoxifen	Sigma-Aldrich	Cat# T5648
**Critical commercial assays**		
RBC Lysis Buffer	Beyotime	Cat# C3702
RIPA Lysis Buffer	Beyotime	Cat# P0013B
CCK-8 Cell Proliferation and Cytotoxicity Assay Kit	Solarbio	Cat# CA1210
His-tag Protein Purification Kit	Beyotime	Cat# P2229S
Omni-ECL™Femto Light Chemiluminescence Kit	Epizyme	Cat# SQ201L
Pierce™ BCA Protein Assay Kit	Thermo Fisher Scientific	Cat# 23227
EasySep™ Mouse CD8+ T Cell Isolation Kit	Stemcell	Cat# 19853
Tumor Dissociation Kit, mouse	Miltenyi Biotec,	Cat# 130-096-730
ATP Assay kit	Beyotime	Cat# S0026
RNAprep Pure Tissue Kit	TIANGEN	Cat# DP431
**Deposited data**		
Single-cell RNA seq	GEO database	GSE215130
**Experimental models: Cell lines**		
CT26	ATCC	Cat# CRL-2638™
4T1	ATCC	Cat# CRL-2539™
B16-F10	ATCC	Cat# CRL-6475™
HEK 239T/17	ATCC	Cat# CRL-11268™

Hepa1-6	ATCC	Cat# CRL-1830™
LLC1	ATCC	Cat# CRL-1642™
**Experimental models: Organisms/strains**		
Mouse: C57BL/6J	GemPharmatech	Strain NO. N00013
Mouse: BALB/c	GemPharmatech	Strain NO. N00020
Mouse: B6.Cg-Tg(Cebpb-Tta)5Bjd/J	The Jackson Laboratory	Strain #: 003563; RRID: IMSR_JAX:003563
Mouse: FVB/N-Tg(tetO-MYC)36aBop/J	The Jackson Laboratory	Strain #: 019376; RRID: IMSR_JAX:019376
Mouse: NOD/ShiLtJGpt-Prkdc^{em26Cd52}Il2rg^{em26Cd22}Hr^{em1Cin8936}/Gpt mice(NCG)	GemPharmatech	Strain #: T003257
Mouse: huCD34^{+}HSC-NOD/ShiLtJGpt-Prkdc^{em26Cd52}Il2rg^{em26Cd22}/Gpt	GemPharmatech	Strain #: T037620
**Recombinant DNA**		
P23-GGA3-FLAG	This paper	N/A
Pet-21+(b)-SEC7-His	This paper	N/A
Pet-21+(b)-Arf1-His	This paper	N/A
Plko.1-shLuciferase-puromycin	This paper	N/A
Plko.1-shArf1-1-puromycin	This paper	N/A
Plko.1-shArf1-2-puromycin	This paper	N/A
Plko.1-shSPHK1-1-puromycin	This paper	N/A
Plko.1-shSPHK1-2-puromycin	This paper	N/A
**Software and algorithms**		
FlowJo_V10	Becton Dickinson	https://www.flowjo.com/solutions/flowjo
GraphPad Prism 9.0.0	GraphPad	https://www.graphpad.com/
AutoDock Tools 1.5.6	Molecular Graphics Laboratory	https://ccsb.scripps.edu/mgltools/
Discovery Studio Visualizer	Dassault Systèmes	https://discover.3ds.com/discovery-studio-visualizer-download
PyMOL	Schrödinger	https://pymol.org/2/
ZEN lite	ZEISS	https://www.zeiss.com
